# Genome-Wide Identification and Expression Profiling of Odorant-Binding Protein Genes in the Bean Flower Thrips *Megalurothrips usitatus* (Bagnall) (Thysanoptera: Thripidae)

**DOI:** 10.3390/insects16020212

**Published:** 2025-02-14

**Authors:** Gen Xia, Lang Yang, Boliao Li, Qinli Wang, Lifei Huang, Xiaoli Tian, Guohui Zhang

**Affiliations:** 1 College of Agriculture, Yangtze University, Jingzhou 434025, China; 2023710835@yangtzeu.edu.cn (G.X.); 13477866904@163.com (Q.W.); 2 Guangxi Key Laboratory of Biology for Crop Diseases and Insect Pests, Key Laboratory of Green Prevention and Control on Fruits and Vegetables in South China Ministry of Agriculture and Rural Affairs, Plant Protection Research Institute, Guangxi Academy of Agricultural Science, Nanning 530007, China; yang2001lang@163.com (L.Y.); hlfhlf@gxaas.net (L.H.); 3 Shaanxi Province Key Laboratory of Jujube, College of Life Science, Yan’an University, Yan’an 716000, China; liboliao@yau.edu.cn; 4 College of Life Science, Yangtze University, Jingzhou 434025, China; lyacxiaoli@163.com

**Keywords:** *Megalurothrips usitatus* (Bagnall), genome-wide identification, odorant-binding protein, expression pattern

## Abstract

*Megalurothrips usitatus* is an economically important vegetable pest. Because of the growing demand for reducing pesticide use on vegetables, new environmentally friendly strategies for controlling *M. usitatus* are urgently needed. Insect odorant-binding proteins are prospective targets for screening environmentally friendly odorant attractants for pest control. However, very little is known about OBP genes in *M. usitatus*. Here, we identified 14 *OBPs* in the *M. usitatus* genome using HMMER and BLAST. The chromosomal location showed that these *OBPs* were widely distributed across eight chromosomes. The analysis of the gene and protein structure characteristics of OBPs in *M. usitatus* revealed substantial diversity within the OBP gene family. The spatiotemporal expression profiles showed that ten out of 14 *MusiOBPs* displayed male biased expression, which were highly expressed in antennae, suggesting that they may play a crucial role in the recognition of host plant volatiles and thrips aggregation pheromones. Notably, only *MusiOBP8* was significantly higher expressed in female adults, indicating a potential involvement in reproduction. Moreover, *MusiOBP7* and *MusiOBP13* were highly expressed in the pupae, indicating their possible role in immune responses. These results provide an important foundation for further exploration of the functions of the OBPs in *M. usitatus*.

## Simple Summary

The bean flower thrips *Megalurothrips usitatus* is an important Leguminosae pest in Asia and has caused enormous economic losses to leguminous crops in Southern China. The current management of this pest is still the application of chemical insecticides and lacks green and efficient control methods. Insect odorant-binding proteins (OBPs) play a central role in discriminating and transporting odorant molecules to odorant receptors, and, thus, OBPs are considered to be key molecular targets for screening environmentally friendly odorant attractants for pest control. Here, we systematically identified 14 OBP genes from the genome of *M. usitatus* and analyzed their basic structural characteristics and spatiotemporal expression profiles for the first time. The findings of this study will help researchers to further investigate the functions of these OBPs and to develop environmentally friendly control methods against *M. usitatus*.

## 1. Introduction

The bean flower thrips, *Megalurothrips usitatus* (Bagnall) (Thysanoptera: Thripidae), is a destructive pest attacking leguminous crops in Asia [1,2,3,4]. The insect can damage the whole growth period of legumes by feeding on flowers, leaves, and pods and laying eggs in plant tissue, resulting in leaf deformity, necrosis and premature abortion of buds and flowers, and pod scab [3,5]. It can cause significant losses of yield, quality, and commodity value in crop production. To manage this pest, applying chemical insecticides is still the main method [6,7,8]. However, excessive pesticide usage against *M. usitatus* has led to the development of resistance and the production of pesticide residues that cause environmental contamination and risk consumer health [9,10,11]. Therefore, developing green and efficient management strategies against *M. usitatus* is important.

Insects have evolved a sensitive olfactory system to locate mates, host plants, and oviposition sites and escape from threats [12,13,14]. It has been demonstrated that the accurate operation of the system relies on a series of olfactory proteins, including odorant-binding proteins (OBPs), chemosensory proteins (CSPs), odorant receptors (ORs), gustatory receptors (GRs), ionotropic receptors (IRs), sensory neuron membrane proteins (SNMPs), odorant degrading enzymes (ODEs), and Niemann–Pick protein C2 (NPC2) [15,16]. Notably, OBPs are involved in the first biochemical reaction in the olfactory transduction cascade and play a central role in discriminating, binding, and transporting odorant molecules to ORs [17,18,19]. OBPs are small, globular, and hydrosoluble proteins [20,21]. The characteristic of OBPs is the highly conserved cysteines (Cys) that are paired to form interlocked disulfide bonds [22]. Based on the number of Cys residues in sequences, OBPs can be divided into four major subfamilies: Classic OBPs (with six conserved Cys residues), Minus-C OBPs (with four or five Cys residues), Plus-C OBPs (at least eight Cys residues and a conserved proline residue after the sixth Cys residue), and Atypical OBPs (with nine or ten Cys residues and an extended C-terminal region) [19,23,24]. Most studies have shown that OBPs could participate in the perception of external odorant cues. For example, mutants of *OBP76a* (lush) show abnormal behavioral responses to *cis*-vaccenyl acetate (cVA), the male sex pheromone in *Drosophila melanogaster* [25]. Similarly, knockdown of *ApisOBP3* and *ApisOBP7* altered the adult repellent behavioral responses towards the alarm pheromone (*E*)-β-farnesene (EBF) in aphids *Acyrthosiphon pisum* [26]. In *Rhynchophorus ferrugineus*, RferOBP8 and RferOBP11 are involved in the discrimination of palm volatiles and aggregation pheromones [27]. Therefore, OBPs have the potential to be used as molecular targets to screen efficient pest behavioral control agents. In addition to odorant perception functions, some OBPs have other physiological functions, including in taste, mating behavior, humidity detection, immunity response, and insecticide resistance [28,29,30,31,32].

Since the first OBP was reported in the male antennae of *Antheraea polyphemus* in 1981 [22], insect OBPs have been gradually identified by transcriptome or genomic analyses. For instance, 51 *OBPs* were identified in the genome of *Drosophila melanogaster* [23], 7 *OBPs* in the antennal transcriptome of *Frankliniella occidentalis* [33], 6 *OBPs* in the antennal transcriptome of *F. intonsa* [33], and 33 *OBPs* in the genome of *Spodoptera frugiperda* [34]. However, the current understanding of the OBPs in *M. usitatus* is insufficient. To date, only one OBP gene has been identified in *M. usitatus* from the transcriptome [35]. Recently, the chromosome-level genome of *M. usitatus* has been released [36], and we can deeply explore OBPs in the *M. usitatus* genome. In this study, we first identified OBP genes in the genome of *M. usitatus*. Then, we analyzed their basic characteristics, including chromosomal location, phylogenetic analysis, gene structure, motif, and domain analysis. Finally, real-time quantitative polymerase chain reaction (RT-qPCR) was used to analyze the expression patterns of these OBP genes in different developmental stages and tissues of both sexes. The results will help us to investigate the functions of OBPs and to develop environmentally friendly control methods against *M. usitatus*.

## 2. Materials and Methods

### 2.1. Identification of OBP Genes in M. usitatus

The whole-genome sequence assembly Mus_1.0 (GenBank assembly number: GCA_026979955.1), GFF annotation file, total CDS sequence, and total protein sequence file of *M. usitatus* were acquired from the NCBI website (https://www.ncbi.nlm.nih.gov/datasets/genome/GCA_026979955.1/, accessed on 18 January 2024). Putative odorant-binding protein (OBP) genes in *M. usitatus* were identified using HMMER and BLAST based on genome data. The HMM file for OBP (PF01395: PBP/GOBP family) was downloaded from the Pfam database (v. 35.0). HMMER searching was conducted using the HMMER software (version 3.3) [37] to retrieve potential protein sequences of *M. usitatus* OBPs. Subsequently, the potential OBPs were manually checked by BLASTP searches against the NCBI Nr database [38]. The final identification results were combined with the results of these two approaches. Putative N-terminal signal peptides of all OBPs were predicted by the SignalP5.0 online program (https://services.healthtech.dtu.dk/services/SignalP-5.0/, accessed on 18 January 2024) [39]. The protein sequences of OBPs without signal peptides were aligned using the muscle method with Jalview software (version 2.11.4.0) [40].

### 2.2. Chromosomal Location, Phylogenetic Analysis, and Structural Characteristics Analyses of MusiOBPs

Chromosomal location information of OBP genes was extracted from the *M. usitatus* GFF annotation file and mapped onto the chromosomes using TBtools (version 2.154) [41]. The phylogenetic tree of full-length protein sequences of OBPs from *M. usitatus* was constructed using the neighbor-joining (NJ) method. Trees with 1000 bootstrap replicates were built and improved using MEGA 11.0 software (version 11.0.13) [42]. The maximum likelihood phylogenetic tree of OBPs was constructed with the full-length protein sequences of OBPs from *M. usitatus* (Thysanoptera), *Odontothrips loti* (Thysanoptera), *Thrips palmi* (Thysanoptera), *F. occidentalis* (Thysanoptera), *F. intonsa* (Thysanoptera), *Locusta migratoria* (Orthoptera), *A. pisum* (Hemiptera), *D. melanogaster* (Diptera), *Tribolium castaneum* (Coleoptera), *Apis mellifera* (Hymenoptera), and *S. exigua* (Lepidoptera) (Supplementary Data Sheet S1) using the IQ-TREE web server (http://iqtree.cibiv.univie.ac.at/, accessed on 28 January 2025) with 1000 bootstrap replicates and visualized in iTOL (https://itol.embl.de/, accessed on 28 January 2025). The exon–intron structure of *MusiOBP* genes was constructed by TBtools (version 2.154) [41]. The motif patterns of MusiOBPs were discovered using the MEME online server (https://meme-suite.org/meme/tools/meme, accessed on 31 October 2024) with the number of motifs as 10 [43], and the conservative domains of MusiOBPs were analyzed by the Conserved Domain Database (NCBI-CDD) (https://www.ncbi.nlm.nih.gov/Structure/bwrpsb/bwrpsb.cgi, accessed on 31 October 2024) [44]. Finally, the above results of structural characteristics were visualized and merged via TBtools (version 2.154) [41].

### 2.3. Insect Rearing and Sample Collection

The insects used in this study were from a laboratory colony of *M. usitatus* provided by the Institute of Plant Protection and Microbiology, Zhejiang Academy of Agricultural Sciences, Hangzhou, China. The population was reared on fresh *Lablab purpureus* in an artificial incubator at 26 ± 1 °C, 70 ± 5% relative humidity, with a photoperiod of 14 L: 10 D. For developmental stage expression profiling, the first instar nymphs (60), second instar nymphs (60), prepupae (60), pseudo-pupae (50), 1–3-days-old female adults (60), and 1–3-days-old male adults (100) were collected separately. For tissue expression profiling, around 150 male and 100 female *M. usitatus* adults of 1–3 days old were dissected on ice to remove the antennae using 1 mL medical syringes under a light microscope. Then, adults without antennae (A−) were obtained and collected. Adults (A+) of male (150) and female (100) of 1–3 days old were collected separately after eclosion. All samples were collected with three biological replicates and stored at −80 °C until use.

### 2.4. RNA Extraction and cDNA Synthesis

Total RNA was extracted from the above samples using M5 Total RNA Extraction Reagent (Mei5bio, Wuhan, China) according to the manufacturer’s instructions. The quality and quantity of the RNA samples were determined using a NanoDrop 2000 spectrophotometer (Thermo Fisher Scientific, Waltham, MA, USA). In addition, the integrity of the RNA was detected with 1% agarose electrophoresis. Subsequently, 1 μg of total RNA from different developmental stages’ samples and 0.5 μg of total RNA from different tissues’ samples were reverse transcribed to cDNA using the PrimeScript RT Reagent Kit with gDNA Eraser (TaKaRa, Dalian, China) following the manufacturer’s protocol, after which the cDNA was stored at −20 °C until use.

### 2.5. Real-Time Quantitative PCR Analysis of MusiOBPs

Real-time quantitative PCR (RT-qPCR) was used to analyze the expression levels of OBP genes in different developmental stages and tissues of both sexes. Gene-specific primers were designed with the Primer3 web service (https://primer3.ut.ee/, accessed on 20 March 2024) and are shown in Table 1. The lengths of the PCR products ranged from 100 to 211 bp. The cDNA templates from different developmental stages and tissues were diluted 5-fold with sterile water, and 1 μL of the diluted cDNA was used as template for subsequent RT-qPCR analysis. RT-qPCR was performed with TB Green^®^ *Premix Ex Taq*™ II (Tli RNaseH Plus) (TaKaRa, Dalian, China) in the ABI QuantStudio^TM^ 6 Flex Real-Time PCR System (Applied Biosystems, Foster City, CA, USA). Each RT-qPCR reaction was conducted in a total volume of 20 μL mixture containing 10 μL of TB Green *Premix Ex Taq* II (Tli RNaseH Plus) (2×), 0.8 μL of each primer (10 μM), 0.4 μL of ROX Reference Dye II (50×), 1 μL of diluted cDNA, and 7 μL of sterile water. The amplification procedure was as follows: 95 °C for 30 s, followed by 40 cycles at 95 °C for 5 s, 51/54 °C for 15 s, and 72 °C for 20 s. After 40 cycles, 95 °C, 15 s, and melting curves were conducted at increments of 0.5 °C from 60 to 95 °C for 0.05 s each to detect the presence of a single gene-specific peak and the absence of primer dimer peaks for each gene. All developmental stages and tissues were performed with three biological and three technical replications. The relative expression levels of OBP genes of *M. usitatus* were analyzed using the 2^−ΔΔCT^ method [45] and normalized with the reference genes *RPL* and *ACT* of *M. usitatus* [46]. The data are represented as the mean ± standard error (mean ± SE). Statistical comparison of gene expression in different developmental stages was performed using a one-way analysis of variance followed by Tukey’s honestly significant difference test. Statistical significance of different letters was considered at *p* < 0.05. The Student’s *t*-test was used to analyse the expression of these OBP genes in different tissues and sexes (* *p* < 0.05; ** *p* < 0.01; *** *p* < 0.001; ns, not significant). The statistical analysis was conducted and visualized on IBM SPSS software (version 26.0) and GraphPad Prism software (version 10.1.2) (Inc., La Jolla, CA, USA).

## 3. Results

### 3.1. Genome-Wide Identification of OBP Genes in M. usitatus

**Figure 1:**
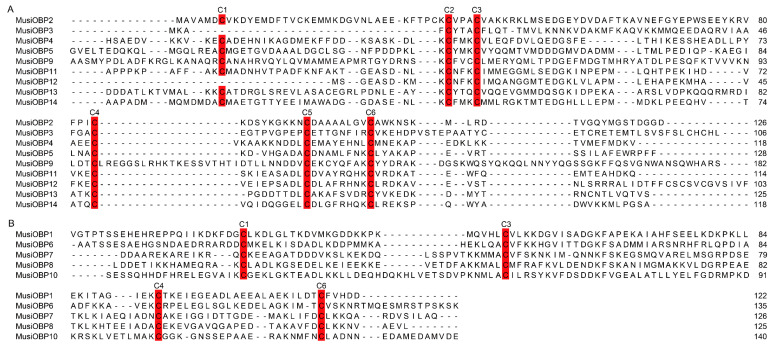
Amino acid sequence alignment of OBPs without signal peptide in *M. usitatus*. (**A**) Alignment of Classic OBPs in *M. usitatus*. (**B**) Alignment of Minus-C OBPs in *M. usitatus*. The conservative cysteine residues (C1–C6) are indicated.

In the present study, 14 OBP genes were identified in *M. usitatus* based on the Mus_1.0 genome data using the Hidden-Markov-Model (HMM) search and BLASTP (Table 2). Based on the chromosomal location, the sequences were named *MusiOBP1-14*. These OBP genes had complete open reading frames (ORFs), potentially encoding proteins consisting of 103 (MusiOBP12) to 211 (MusiOBP9) amino acids, with the majority falling around the 140 amino acid mark (Table 2). Most of these OBPs had a signal peptide ranged from 17 to 29 amino acids, except for MusiOBP2, 3, 12 (Table 2). In addition, most of the MusiOBPs had homology with the OBPs of *F. fusca* and *O. loti* (Table 2). Based on the number of conserved cysteines, the OBPs of *M. usitatus* were divided into two subfamilies, namely, Classic OBPs and Minus-C OBPs. Nine OBPs of *M. usitatus* belonged to the Classic OBP subfamily (MusiOBP2-5, MusiOBP9, and MusiOBP11-14), which had six conserved cysteine residues and corresponding spaces (Figure 1A). Among them, MusiOBP3 and MusiOBP12 lacked the conserved C1, although classified as a Classic OBP given the conservation of the other five conserved cysteines and corresponding spaces (Figure 1A). The remaining five OBPs of *M. usitatus* belonged to the Minus-C OBP subfamily (MusiOBP1, MusiOBP6-8, and MusiOBP10), which had four conserved cysteine residues, lacking C2 and C5 (Figure 1B).

### 3.2. Chromosomal Location of OBP Genes in M. usitatus

**Figure 2:**
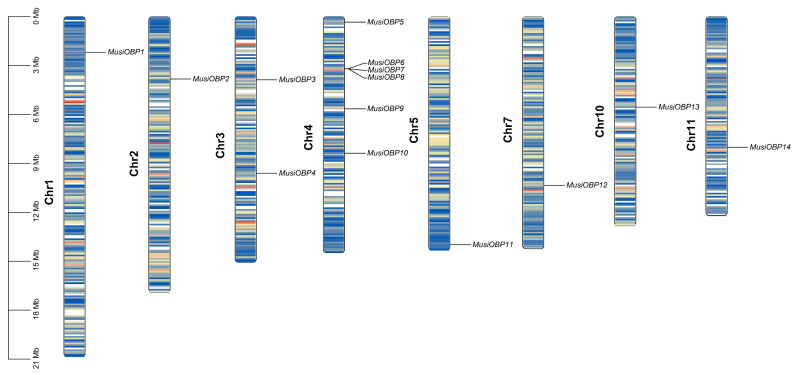
Chromosome localization of the OBP genes in the *M. usitatus* genome.

The chromosomal location analysis of all 14 *OBPs* showed that they were widely distributed across eight out of the total sixteen chromosomes, with *OBPs* located on Chr1-5, Chr7, Chr10, and Chr11 (Figure 2). Notably, Chr4 harbored six *OBPs*, and three *OBPs* (*MusiOBP6-8*) clustered tightly on Chr4 (Figure 2). Additionally, Chr3 harbored two *OBPs*, while Chr1, Chr2, Chr5, Chr7, Chr10, and Chr11 each harbored a single *OBP* (Figure 2).

### 3.3. Gene and Protein Structure of OBPs in M. usitatus

**Figure 3:**
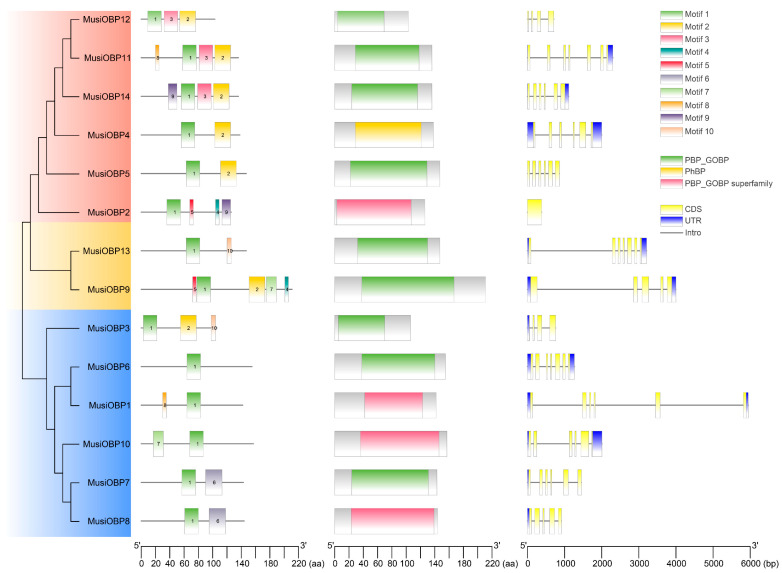
Phylogenetic relationships, conservative motifs, domains, and gene structure analysis of OBPs in *M. usitatus*. Red, subgroup 1; yellow, subgroup 2; and blue, subgroup 3.

The analysis of the motif pattern and conservative domains of OBP proteins and exon-intron of OBP genes in *M. usitatus* revealed substantial diversity within the OBP gene family (Figure 3). The phylogenetic tree of OBPs in *M. usitatus* divided the full-length protein sequences into three subgroups. Motif pattern analysis revealed that all OBP sequences presented motif 1, and the OBP sequences from subgroup 1 presented motif 1 and motif 2, except for MusiOBP2 (Figure 3). Notably, MusiOBP9 from subgroup2 had the most motifs (five motifs), while MusiOBP6 from subgroup3 presented only motif 1 (Figure 3). The conservative domain analysis using NCBI-CDD confirmed that all OBPs contained PBP_GOBP and PhBP domains (Figure 3). In addition, the exon–intron analysis showed that the number of exons ranged from one to seven, with most *OBPs* containing six to seven exons (Figure 3). Notably, *MusiOBP2* contained only a long exon. Additionally, eight *OBPs* exhibited 5′ and 3′ UTRs, three genes (*MusiOBP3*, *MusiOBP7*, and *MusiOBP8*) contained only 5′ UTR, while three others (*MusiOBP2*, *MusiOBP5*, and *MusiOBP12*) presented no UTR (Figure 3).

### 3.4. Phylogenetic Analysis of OBPs

**Figure 4:**
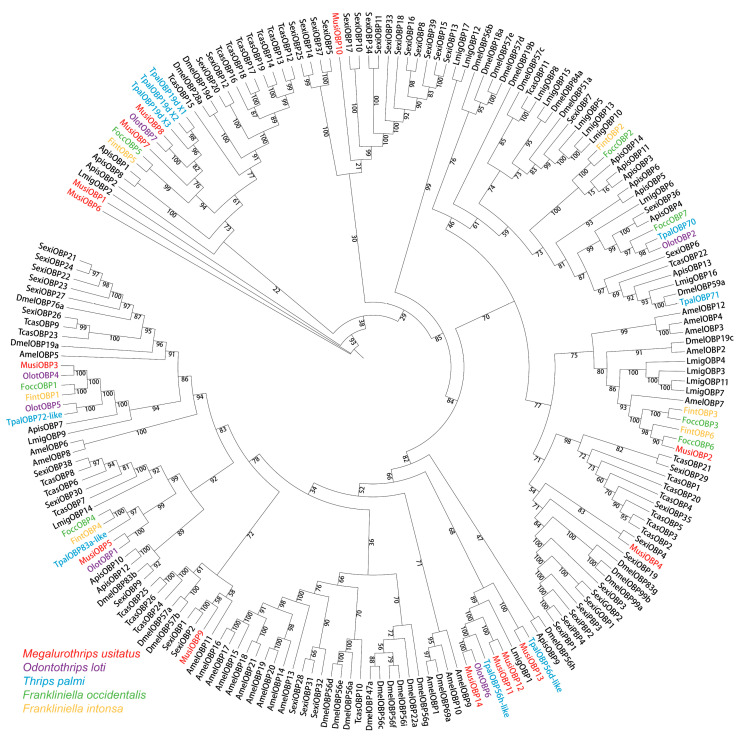
Maximum likelihood tree of OBPs from *M. usitatus* and other ten species from seven insect orders. Musi: *Megalurothrips usitatus* (Thysanoptera); Olot: *Odontothrips loti* (Thysanoptera); Tpal: *Thrips palmi* (Thysanoptera); Focc: *Frankliniella occidentalis* (Thysanoptera); Fint: *Frankliniella intonsa* (Thysanoptera); Lmig: *Locusta migratoria* (Orthoptera); Apis: *Acyrthosiphon pisum* (Hemiptera); Dmel: *Drosophila melanogaster* (Diptera); Tcas: *Tribolium castaneum* (Coleoptera); Amel: *Apis mellifera* (Hymenoptera); Sexi: *Spodoptera exigua* (Lepidoptera). Bootstrap values (%) based on 1000 replicated are indicated.

To show the homologous relationships of all OBPs in *M. usitatus* with other insect OBPs, a phylogenetic tree was established using the full-length protein sequences of 196 OBPs of 11 species from seven orders (Figure 4). In terms of species affinities, nine out of fourteen MusiOBPs were more closely related to Thysanoptera, especially to *O. loti*, which was consistent with the results of BLASTP (Table 2). Among them, MusiOBP2 was a homolog of FoccOBP6; MusiOBP3 was a homolog of OlotOBP4; MusiOBP5 was a homolog of OlotOBP1; MusiOBP8 was a homolog of OlotOBP7; MusiOBP11 and MusiOBP12 were closely related to TpalOBP56h-like; MusiOBP13 was a homolog of TpalOBP56d-like; and MusiOBP14 was a homolog of OlotOBP6 (Figure 4). Interestingly, three MusiOBPs had homology with the OBPs of *S. exigua*. MusiOBP4 was closely related to SexiOBP4; MusiOBP9 was closely related to SexiOBP1 and SexiOBP2; and MusiOBP10 was closely related to SexOBP10 and SexiOBP17 (Figure 4).

### 3.5. Expression Patterns of OBPs in Different Developmental Stages of M. usitatus

**Figure 5:**
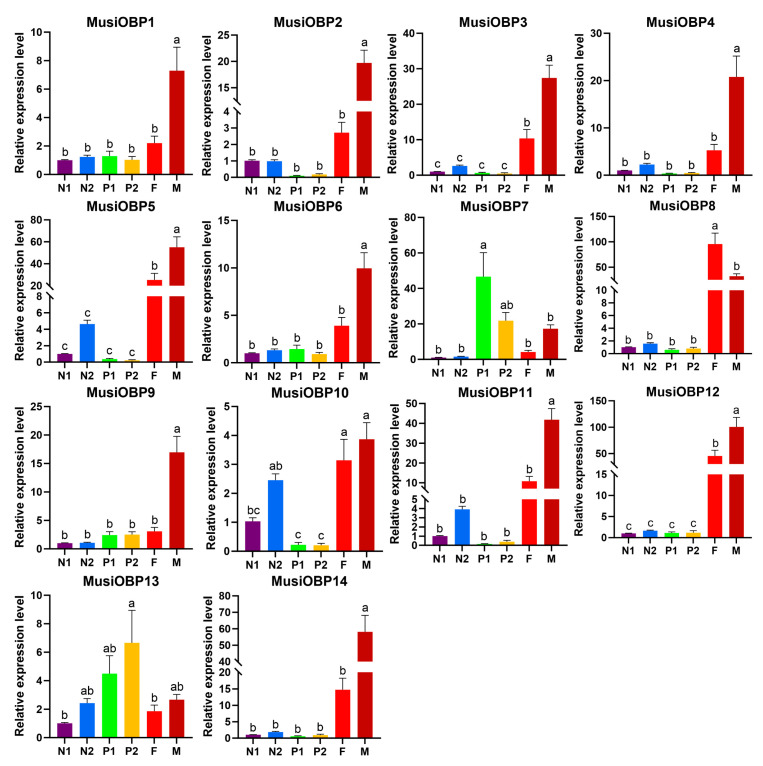
Expression profile of OBP genes in different developmental stages of *M. usitatus* by RT-qPCR. The first instar nymphs (N1) are taken as the normalized sample. Different lowercase letters indicate significant differences (*p* < 0.05) by using a one-way analysis of variance followed by Tukey’s honestly significant difference test. Standard errors are indicated by error bars. N1: first instar nymphs; N2: second instar nymphs; P1: prepupae; P2: pseudo-pupae; F: female adults; M: male adults.

The results of the relative expression level of *OBPs* showed that ten out of fourteen *OBPs* (*MusiOBP1-6*, *MusiOBP9*, *MusiOBP11*, *MusiOBP12*, and *MusiOBP14*) were significantly higher expressed in male adults, while *MusiOBP8* was significantly higher expressed in female adults (Figure 5). Interestingly, *MusiOBP10* was highly expressed in both female and male adults, followed by the second instar nymphs (N2), but there were no significant differences between the three of them (Figure 5). In addition, *MusiOBP7* was highly expressed at the prepupae stage (P1), while *MusiOBP13* was highly expressed at the pseudo-pupae stage (P2) (Figure 5).

### 3.6. Tissue- and Sex-Specific Expression Patterns of OBPs in M. usitatus

**Figure 6:**
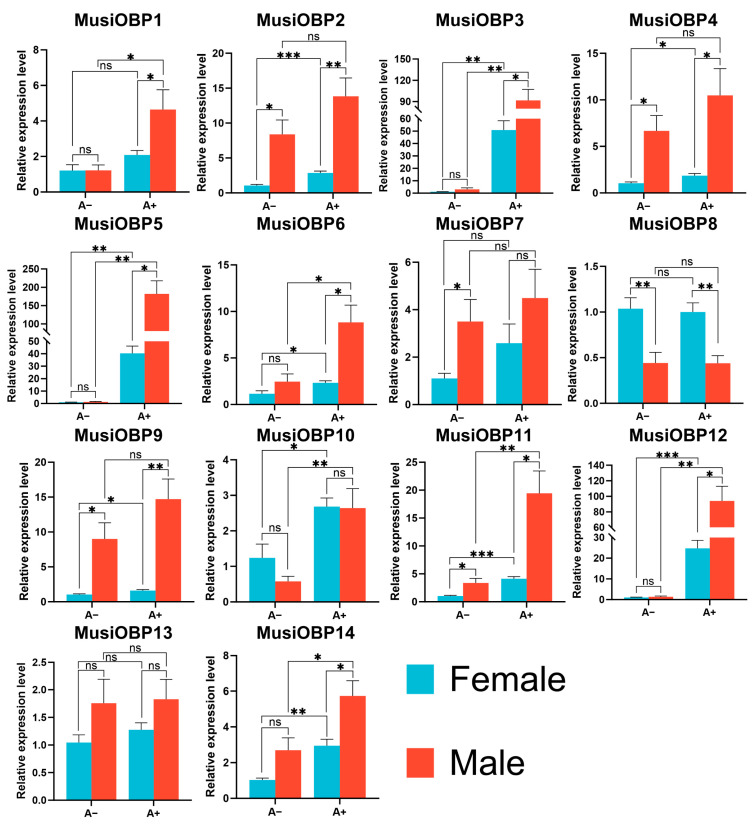
Expression profile of OBP genes in different tissues of *M. usitatus* by RT-qPCR. The female adults without antennae are taken as the normalized sample. Asterisks indicate significant differences in different tissues and sexes by using Student’s *t*-test (* *p* < 0.05; ** *p* < 0.01; *** *p* < 0.001; ns, not significant). Standard errors are indicated by error bars. A−: adults without antennae; A+: adults with antennae.

The expression of 14 *OBPs* in adults (A+) and adults without antennae (A−) of both sexes in *M. usitatus* were monitored by RT-qPCR (Figure 6). The results showed that 11 out of 14 *OBPs* had significantly different expression levels between the same sex adults with and without antennae (*p* < 0.05), except for *MusiOBP7*, *MusiOBP8*, and *MusiOBP13* (Figure 6). Among them, seven *OBPs* (*MusiOBP3*, *MusiOBP5*, *MusiOBP6*, *MusiOBP10-12,* and *MusiOBP14*) were expressed significantly higher in adults (A+) of both sexes, and three *OBPs* (*MusiOBP2*, *MusiOBP4*, and *MusiOBP9*) were expressed significantly higher in female adults (A+), while *MusiOBP1* was expressed significantly higher in male adults (A+) (Figure 6). In addition, the results also showed that 12 out of 14 *OBPs* had significantly different expression levels in different sexes of adults with/without antennae (*p* < 0.05), except for *MusiOBP10* and *MusiOBP13* (Figure 6). Among them, five *OBPs* (*MusiOBP2*, *MusiOBP4*, *MusiOBP8*, *MusiOBP9*, and *MusiOBP11*) showed significant differences in adults (A+) and adults without antennae (A−) between females and males, and six *OBPs* (*MusiOBP1*, *MusiOBP3*, *MusiOBP5*, *MusiOBP6*, *MusiOBP12*, and *MusiOBP14*) showed significant differences in adults (A+) between females and males, while *MusiOBP7* showed significant differences in adults without antennae (A−) between females and males (Figure 6). Notably, only *MusiOBP8* had a significantly higher expression level in females than males across the adults with/without antennae (Figure 6). After excluding the adults without antennae (A−), we compared the expression levels of 14 *OBPs* in the antennae between females and males. The results showed that nine out of fourteen *OBPs* were expressed significantly higher in male antennae than female antennae (*p* < 0.05), except for *MusiOBP4*, *MusiOBP7*, *MusiOBP8*, *MusiOBP10*, and *MusiOBP13* (Figure S1).

## 4. Discussion

*M. usitatus* is one of the most serious pests of cowpea in China, especially the southern regions [47]. Due to the excessive use of chemical pesticides against *M. usitatus*, coupled with growing concerns about insecticide resistance and food safety, it is essential to develop efficient and sustainable management strategies to control this pest. Insects largely rely on their chemosensory system to survive and reproduce, with OBPs playing a vital role in these chemosensory processes [27,48]. Therefore, a better understanding of the chemosensory system, especially OBPs in *M. usitatus*, could contribute to exploring environmentally friendly control approaches. Despite the important role and potential value of OBP genes, studies on this gene family of *M. usitatus* are rarely reported. In this study, to expand our knowledge of the thrips chemosensory system, we systematically identified *OBP* genes in *M. usitatus* using genome data. Furthermore, we characterized the expression patterns of these *OBPs* in different developmental stages and tissues of both sexes.

The identification of olfactory genes is a pre-requisite for illustrating the molecular basis of chemosensation [49]. OBP genes have been identified in numerous insect species using transcriptome and genome analyses [23,24,50,51]. The number of OBP genes varies considerably across species, ranging from 7 in *Megachile rotundata* to 109 in *Cluex quinquefasciatus* [52]. In the present study, we identified 14 OBP genes in *M. usitatus* genome, higher than that observed in other thrip species, such as *F. occidentalis* (twelve *OBPs*) [53], *T. palmi* (eight *OBPs*) [53], *F. intonsa* (six *OBPs*) [33] and *O. loti* (six *OBPs*) [53], which may be associated with its higher environmental adaptability, especially on cowpea *Vigna unguiculata* L. [54]. In addition, the sequence of *MusiOBP5* in our findings is similar to *MusiOBP1* identified by Li et al. [35], but the 3′ end differs. This discrepancy is likely due to errors occurring during the amplification or splicing process, as *MusiOBP1* was obtained by using RACE PCR.

Chromosomal location, gene structure, and conserved domain analysis are crucial for exploring the evolutionary relationships and functional characteristics of OBPs [55,56]. Our results showed that most of *MusiOBPs* were distributed on different chromosomes, with six *OBPs* clustered on Chr4, followed by two *OBPs* on Chr3. This distribution suggests potential gene duplications in the genome [57], which may indicate a relatively recent expansion of the OBP genes of *M. usitatus* [58]. Notably, three of them were clustered tightly on Chr4, which may have analogous functions [34]. For example, in *Solenopsis invicta*, ten of twenty-four *OBPs* located in the social chromosome may involve in regulating behavioral differences between single- and multiple-queen colonies [59]. In addition, our results showed that the OBP genes in *M. usitatus* had divergent patterns of exon–intron organization, which may broaden a potential for alternative splicing [34] and contribute to functional diversification [58]. Moreover, the motif analysis further highlighted the variable motif patterns, suggesting that these OBPs may have specialized and divergent functions. Nevertheless, all MusiOBPs contained PBP_GOBP and PhBP domains, which are characteristic features of insect OBP [60].

Phylogenetic analysis among 11 species from seven orders showed that the OBPs from *M. usitatus* were more closely related to OBPs from *O. loti*, *S. exigua*, *T. palmi,* and *F. occidentalis*, which share some host plants with *M. usitatus* [33,53,61], indicating that these phylogenetically correlated OBPs may have similar functions. Notably, MusiOBP2 and MusiOBP14 were closely related to FoccOBP6 and OlotOBP6, respectively. In *F. occidentalis*, FoccOBP6 may involve in delivering two aggregation pheromones [33], while OlotOBP6 in *O. loti* plays a role in host plant location [53]. Thus, MusiOBP2 and MusiOBP14 may have similar roles.

The expression patterns provide important cues for exploring their functions. In this study, the expression patterns in different developmental stages showed that 12 out of 14 *OBPs* (except for *MusiOBP7* and *MusiOBP13*) were highly expressed in adults (Figure 5) and 11 in antennae out of 12 *OBPs* (Figure 6, *MusiOBP1-6*, *MusiOBP9-12*, and *MusiOBP14*), especially in male adults (Figure 6, *MusiOBP1*, *MusiOBP3*, *MusiOBP5*, *MusiOBP6*, *MusiOBP10-12*, and *MusiOBP14*), implying that these genes may play a crucial role during the adult stage. Similar expression patterns have been reported in other thrips. For instance, five out of seven *OBPs* in *F. occidentalis* and five out of six *OBPs* in *F. intonsa* were highly expressed both in the adult stage and in antennae, suggesting their involvement in host plant volatile recognition and thrips aggregation pheromones detection [33]. Moreover, in *O. loti*, *OBP1*, *OBP4,* and *OBP6* were highly expressed both in male adults and in antennae, with *OBP6* specifically implicated in host location [53]. Thus, these 12 *OBPs* of *M. usitatus* may be associated with a role in olfaction, such as host plant location, mating, and reproduction. Interestingly, only *MusiOBP8* was significantly higher expressed in female adults, which even may not express in antennae, suggesting that this *OBP* may play an important role in female-specific behaviors, such as reproduction.

*MusiOBP10* was highly expressed not only in adults but also in the second instar nymphs (N2). Previous studies have documented OBP expression in both adults and nymphs, with findings indicating that these *OBPs* may mediate the response to alarm pheromones [62]. In *M. usitatus*, male adults can produce an aggregation pheromone that attracts conspecific female and male adult individuals [63], while *Dendrothrips minowai* larvae release two aggregation pheromones to attract larvae and adults [64]. Hence, *MusiOBP10* may participate in the detection of aggregation pheromones.

*MusiOBP7* and *MusiOBP13* were highly expressed in the prepupae and pseudo-pupae stage, respectively. Recently, a study found that *PverOBP18* was highly expressed in the pupae, which enhanced the pathogen resistance of *Plagiodera versicolora* larvae [65]. Therefore, *MusiOBP7* and *MusiOBP13* may play a vital role in immune responses.

The results of tissue expression patterns showed that 11 out of 14 *OBPs* (except for *MusiOBP7*, *MusiOBP8*, and *MusiOBP13*) had significantly different expression levels between the same-sex adults with and without antennae (*p* < 0.05), suggesting that these genes were likely to express in antennae of adults. Antennae are primary olfactory organs for insects, and numerous studies indicate that the highly expressed genes in antennae play a pivotal role in olfaction [66,67,68,69,70]. Thus, these 11 *OBPs* of *M. usitatus* may have potential involvement in olfactory perception, while the remaining three *OBPs* (*MusiOBP7*, *MusiOBP8*, and *MusiOBP13*) appear to serve other physiological functions, as no significant expression differences were observed across the adults with/without antennae. Furthermore, the expression of 12 *OBPs* (except for *MusiOBP10* and *MusiOBP13*) in different sexes of adults with/without antennae exhibited significant differences, implying functional differentiation between females and males. To sum up, these *OBPs* are worth studying for their functions in the future.

## 5. Conclusions

In summary, we systematically identified 14 OBP genes from the whole genome of *M. usitatus* and named the MusiOBPs based on the chromosomal location for the first time. Then, we analyzed their basic characteristics including phylogenetic analysis, gene structure, motif pattern and conservative domain, and spatiotemporal expression profiles. These findings will provide a solid foundation for future research to identify the roles of MusiOBPs, which will also contribute to understanding the olfactory mechanism of *M. usitatus* and develop environmentally friendly control approaches against *M. usitatus* in the future.

## Figures and Tables

**Table 1:** Primer information used for RT-qPCR.

Primer Name	Primer Sequence (5′-3′)	Length (bp)
qMusiOBP1	F: AAAAGCCCAAGATGCAGGTG	162
	R: TGCACTTTTCAATCCCAGCC	
qMusiOBP2	F: GCAAGGAGATGATGAAGGAC	182
	R: CTTGTACTCCTCGGACCAG	
qMusiOBP3	F: AGAACAATAAAGTGGACGCC	160
	R: TTTACGCAGCGAATGAAGTT	
qMusiOBP4	F: CGAGCTGACCCACATCAA	155
	R: TCAACTTGTCCTCTGGCG	
qMusiOBP5	F: GAGCTCACAGAGGACCAGAA	153
	R: CTGGTACACGCACTTCATGT	
qMusiOBP6	F: ACGATCCCATGATGAAGGCA	160
	R: AATCAGCAGCTATGTCCGGT	
qMusiOBP7	F: AGGCGAGGGAAATCAAGAGG	174
	R: TCGACCCTTCCTTGGAAAACT	
qMusiOBP8	F: TAAAGAAGCACGCGATGGAG	158
	R: CTCGTCCAGCACTTTGAAGG	
qMusiOBP9	F: CGACGATGTGTGCGAGAAAT	188
	R: ATGACCTTGCATGCCATTGG	
qMusiOBP10	F: AAGAACATGCTGGCCTGT	211
	R: ACATGTTCTTGGCCCGTT	
qMusiOBP11	F: CACCACCTCCAAAACCAGC	160
	R: CCATCTTCGGACAACATGCC	
qMusiOBP12	F: GCAACTTCAAGTGCATCATG	100
	R: GCGTGCATTTTCTCAGGT	
qMusiOBP13	F: CCGACAATTTGGAGGCCTAC	153
	R: ACTTTGTGGCAATGTCTCGC	
qMusiOBP14	F: TGCAAATGGATATGGACGCC	162
	R: CGAGTAGTAGGTGACCGTCC	
qMusiACT	F: ACGACGTACAACTCCATCAT	125
	R: GTAATCTCCTTCTGCATCCTGT	
qMusiRPL	F: ACATCGAGCTGGGTACTG	122
	R: CACCACCATTTACTGAGCAT	

F: forward primer; R: reverse primer.

**Table 2:** Summary of odorant-binding proteins (OBPs) sequences identified in the genome of *M. usitatus*.

Gene Name	Gene ID	ORF (aa)	Complete ORF	Signal Peptide	Homology Search with Known Proteins				
					Species	Acc. Number	*E*-Value	Identity	Coverage
MusiOBP1	ONE63_000073	142	Yes	1–20	*Nezara viridula*	QCZ25102.1	0.01	48.98%	34%
MusiOBP2	ONE63_005245	126	Yes	No	*Frankliniella intonsa*	WBW64304.1	5 × 10^−75^	88.71%	98%
MusiOBP3	ONE63_006116	106	Yes	No	*Odontothrips loti*	WBU77197.1	6 × 10^−43^	87.65%	76%
MusiOBP4	ONE63_006437	138	Yes	1–20	*Frankliniella fusca*	KAK3931445.1	5 × 10^−32^	50.00%	92%
MusiOBP5	ONE63_006734	147	Yes	1–19	*Odontothrips loti*	WBU77195.1	1 × 10^−77^	88.64%	89%
MusiOBP6	ONE63_006862	155	Yes	1–20	*Macrosteles quadrilineatus*	XP_054263813.1	0.22	25.64%	71%
MusiOBP7	ONE63_006863	143	Yes	1–17	*Frankliniella occidentalis*	XP_026276672.1	5 × 10^−74^	79.56%	95%
MusiOBP8	ONE63_006864	144	Yes	1–19	*Odontothrips loti*	WBU77200.1	1 × 10^−54^	80.33%	84%
MusiOBP9	ONE63_006989	211	Yes	1–29	*Frankliniella fusca*	KAK3918161.1	2 × 10^−90^	67.51%	91%
MusiOBP10	ONE63_007105	157	Yes	1–17	*Zophobas morio*	XP_063907370.1	0.86	29.00%	63%
MusiOBP11	ONE63_008094	136	Yes	1-22	*Frankliniella fusca*	KAK3914559.1	6 × 10^−39^	48.12%	97%
MusiOBP12	ONE63_009400	103	Yes	No	*Frankliniella fusca*	KAK3914559.1	5 × 10^−33^	66.27%	80%
MusiOBP13	ONE63_001264	147	Yes	1–22	*Thrips palmi*	XP_034232292.1	1 × 10^−31^	50.85%	78%
MusiOBP14	ONE63_002028	136	Yes	1–18	*Odontothrips loti*	WBU77199.1	2 × 10^−81^	86.03%	100%

## Data Availability

The entire *M. usitatus* genome data were downloaded from the National Center for Biotechnology information (NCBI) GenBank website (accession number: GCA_026979955.1).

## Supplementary Materials

The following supporting information can be downloaded at: https://www.mdpi.com/article/10.3390/insects16020212/s1, Supplementary Data Sheet S1: The OBP amino acid sequences used for the phylogenetic tree; Figure S1. The relative expression level of OBP genes in the antennae of *M. usitatus*.
