# Supplementary material for: Genome-Wide Identification and Expression Profiling of Odorant-Binding Protein Genes in the Bean Flower Thrips Megalurothrips usitatus (Bagnall) (Thysanoptera: Thripidae)
Source: Insects. 2025 Feb 14;16(2):212. doi: 10.3390/insects16020212 (PMC11856683; doi:10.3390/insects16020212)
Supplement: Supplementary file 1 [file insects-16-00212-s001.zip › Supplementary Files/Figure S1.pdf]

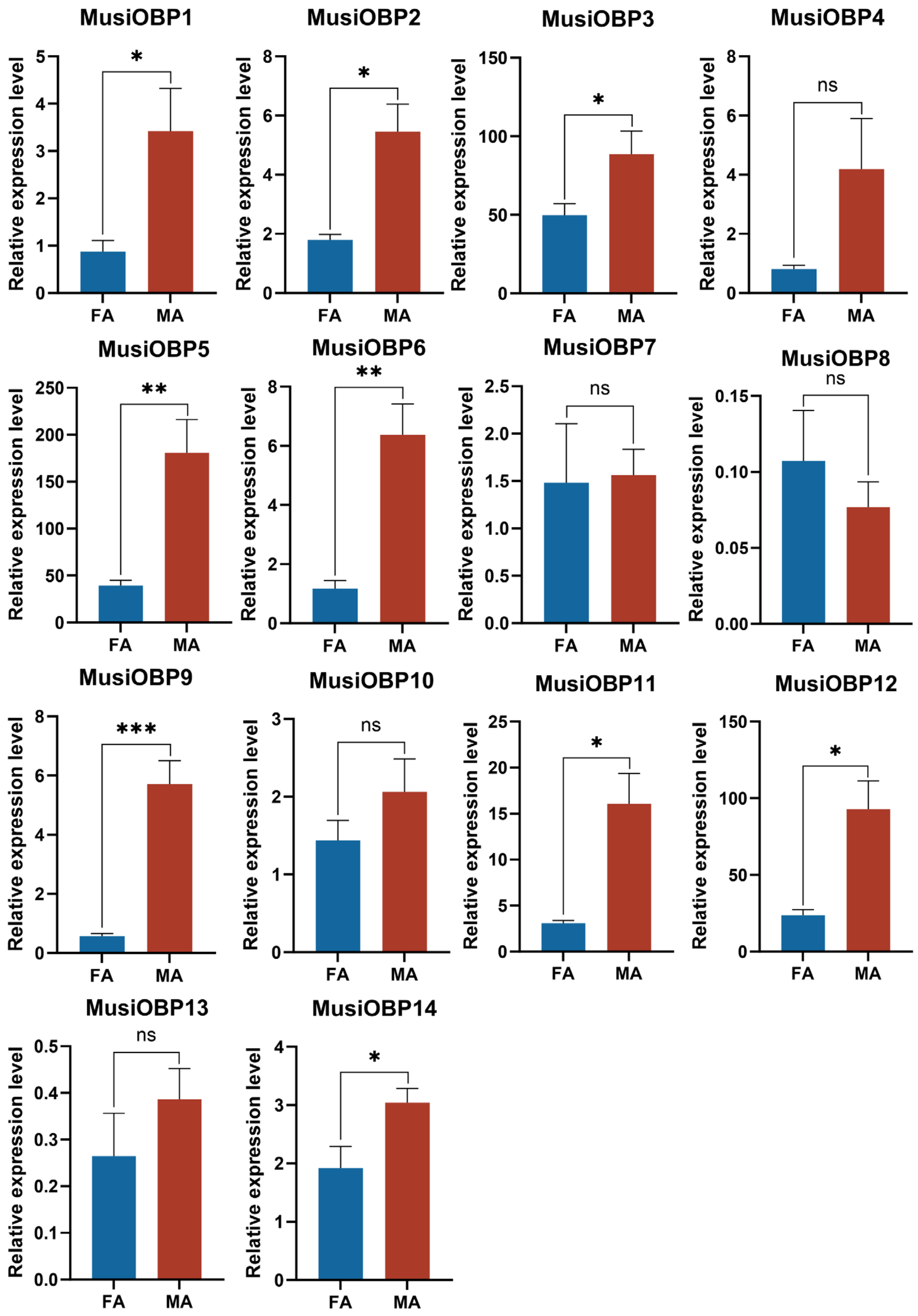

Figure S1. The relative expression level of OBP genes in the antennae of *M. usitatus*. The female adults without antennae are taken as the normalized sample (Figure 6). Asterisks indicate significant differences in the antennae between females and males by using student's *t*-test (* $p < 0.05$; ** $p < 0.01$; *** $p < 0.001$; ns, not significant). Standard errors are indicated by error bars. FA: female antennae, MA: male antennae. The relative expression of 14 *OBPs* in the antennae were obtained using the relative expression in the adults (A+) minus the adults without antennae (A-).
