# Supplementary material for: Genome-Wide Identification and Expression Profiling of Odorant-Binding Protein Genes in the Bean Flower Thrips Megalurothrips usitatus (Bagnall) (Thysanoptera: Thripidae)
Source: Insects. 2025 Feb 14;16(2):212. doi: 10.3390/insects16020212 (PMC11856683; doi:10.3390/insects16020212)
Supplement: Supplementary file 1 [file insects-16-00212-s001.zip › Supplementary Files/Supplementary Data Sheet S1 The OBP amino acid sequences used for the phylogenetic tree.pdf]

>MusiOBP1
MQRALALLLAAAALCGPALAVGTPTSSEHEHREPPQIIKDKFDGCLKDLGLTKDVMKGDD
KKPKMQVHLCVLKKDGVISADGKFAPEKAIAHFSEELKDKPKLLEKITAGIEKCTKEIEGEA
DLAEEALAEKILDTCFVHDD

>MusiOBP2
MAVAMDCVKDYEMDFTVCKEMMKDGVNLAEEKFTPCKCVPACVAKKRKLMSEDGEYD
VDAFTKAVNEFGYEPWSEEYKRVFPICKDSYKGKKNCDAAAALGVCAWKNSKMLRDTVG
QYMGSTDGGD

>MusiOBP3
MKAFCYTACFLQTTMVLKNNKVDAKMFKAQVKKMMQEEDAQRVIAAFGACEGTPVGP
EPCETTGNFIRCVKEHDPVSTEPAATYCETCRETEMTLSVSFSLCHCHL

>MusiOBP4
MDFFPCIALCLLLTLEAALGHSAEDVKKVKECADEHNIKAGDMEKFFDDKSASKDLKCFM
KCVLEQFDVLQEDGSFELTHIKESSHEADLLPYAEECVKAAKKNDDLCEMAYEHNLCMNE
KAPEDKLKKTVMEFMDKV

>MusiOBP5
MNTLLCLAGLLAVCGLASAGVELTEDQKQLMGQLREACMGETGVDAAALDGCLSGNFP
DDPKLKCYMKCVYQQMTVMDDDGMVDADMMLTMLPEDIQPKAEGILNACKDVHGAD
ACDNAMLFNKCLYAKAPEVRTSSILAFEWRPFF

>MusiOBP6
MRVFSVALLVLAAACGTAYCAATSSESAEHGSNDAEDRRARDDCMKELKISDADLKDDPM
MKAHEKLQACVFKKHGVITTDGKFSADMMIARSNRHFRLQPDIAADFKKAVEKCRPELE
GLSGLKEDELAGKIMTCVSKNRTMQESMRSTPSKSK

>MusiOBP7
MKVLVLLAVSLLVCVLADDAAREKAREIKRQCKEEAGATDDDVKSLKEKDQLSSPVTKKM
MACVFSKNKIMQNNKFSKEGSMQVARELMSGRPDSETKLKIAEQIADNCAKEIGGIDTTG
DEMAKLIFDCLKKQARDVSILAQ

>MusiOBP8
MKFYVAIAVAALAAAAVSALDDETIKKHAMEQRAKCLADLKGSEDELKEIEEKKEVETDFA
KKMALCMFRAFKVLDENDKFSKANIMGMAKKVLDGRPEAETKLKHTEEIADACEKEVGA
VQGAPEDTAKAVFDCLKKNVAEVL

>MusiOBP9
MGVPDVAVLRGALIAVLVATLEAPTPVRAAASMYPDLADFKRGLKANAQRCANAHRVQY
LQVMAMMEAPMRTGYDRNSKCFVCCLMERYQMLTPDGEFMDGTMHRYATDLPESQFKT
VVVKNLDTCLREGGSLRHKTKESSVTHTIDTLLNNDDVCEKCYQFAKCYYDRAKDGSKW
QSYQKQQLNNYYQGSSGKFFQSVGNWANSQWHARS

>MusiOBP10
MKVLIVLSTALLAAAAASESSQHHDFHRELEGVAIKCGEKLGKTEADLKKLLDEQHDQKH
LVETSDVPKNMLACILRSYKVFDSDDKFVGEALATLLYELFGDRMPKDKRSKLVETLMAKC
GGKGNSSEPAAERAKNMFNCLADNNEDAMEDAMVDE

>MusiOBP11
MSTMQQALVLVSVLAVVLQMHAAPPPKPAFFAKCMADNHVTPADFKNFAKTGEASDNL
KCNFKCIMMEGGMLSEDGKINPEPMLQHTPEKIHDVVKECSKIEASADLCDVAYRQHKCV
RDKATEWFQEMTEAHDKQ

>MusiOBP12
MSGEASDKMKCNFKCIMQANGGMTEDGKLVLAPMLEHAPEKMHAVFKECVEIEPSADLC
DLAFRHNKCLRDKAVEVSTNLSRRRALIDTFFCSCSVCGVSIVF

>MusiOBP13
MKLAAAALLSAAAALLLAAAHADDDATLKTVMALKKCATDRGLSREVLASACEGRLPD
NLEAYKCYTQCVQQEVGMMDQSGKIDPEKAARSLVDPKQQQRMRDIATKCPGDDTTDLC
AKAFSVDRCYVKEDKDMYTRNCNTLVQTVS

>MusiOBP14
MFAIVVLALLGAQQLAWAAAPADMMQMDMDACMAETGTTYEEIMAWADGGDASENL
KCFMKCMMLRGKTMTEDGHLLLEPMMDKLPEEQHVTATQCVQIDQGGELCDLGFRHQK
CLREKSPQWYADWVKKMLPGSA

>OlotOBP1
MKTILCLAGLLAVCSLASAGVELTEDQKALMKQLREACVGETGVAEAAIDGCLSGNFADD
PKLKCYMKCVYQQMTVMDDDGLVDADMMLTMLPEEIQPKAEPILMACKDVHGSDACD
NAMLFNKCLYAKAPEYYLLP

>OlotOBP2
MKVLVVAVLTVLAVGAANAATPLIRQRCGTPPAAPIRIEKAINECLEDIKLSLDEALSELDAS
AALVSKEVKGRQPGKSRAKRQTFSEDERSIAGCFMHCVHKKAKALNADGVPTGEGLVKLY
AEGSQDANFFLATAQAVNHCMGTAQQQGRLLPQALKEPEQQCYLAYDLFECITDQVVEY
CGTSP

>OlotOBP4
MVHASLLLPLAVLAVAFLDSSSGLPRPAPAPVTQEQYEKSVKMLRNVCQPKTNTPDDILQR
MVDGEFVDDMKAFCYTACFLQTTMVLKNNKVDAKMFKKQVKKMMLEEDAQRVIAAFT
ACESTPVGPEPCQTTANFIRCVKEHDPTFRIPA

>OlotOBP5
METVADSLLCFTTKAAQKMAVYGRVSVLLIGVAALLSSAVHSLPAETEEEMMRAVKSVRAI
CQPKSGATD

EQLTLLSQGSITEARELKCYIGCVLVSYDTLTDGKVDQDKMRALAEKMPESKKETFLLILSKC
KDAGGADECEVGYNYITCAKKTVGEVLHTSFVSRSHSVCTRFCSATHNQHLFSISEILLPCL
REPLQSSALPIIGKLCKSYNKDIPAF

>OlotOBP6
MFAFVALALLSAQQVAWAAAPADMMQMDMDACMAETGTTYEEIMAWADGGDASENL
KCMTKCMMLRCKTMTEDGHLILEAMLAKLPEEQHNIATQCVQIDQGGELCDLAYRHHK
CMRDKSPQWYADWVKKMLPGSS

>OlotOBP7
MKVLIAIAVVAVLVAAVHAVDDDEMKKHALEQRQKCLADLKGDEAELKELEEKHEVTTD
FAKKMTLCMFRAFKIVDENDKYSKANIMEIAKKVLDGKPESEKKLKVAEEIADACDKEVG
ALQGAPEDTAKAVFDCLKKNVVEKTSGVRYGFAYESRAR

>TpalOBP56d-like
MKLAVAALAVLLAAALVAADAEADATAAVQRALKKCQETSKLSVDQLNQACMSTLPSDE
TQKRAYKCFAKCVQQRVGIMSEEGKIDPERSRALVHPSQQEQMKAIAEKCLGDGETDLCEK
AYKVDQCYNKENEKMYQENCKNLIRTITKEA

>TpalOBP56h-like
MLAVVLLAVLCGQQVFGAATASTAATQMPDMHEIMEMCMTETGTSMDDVLSWADGGD
ASENTKCQMKCVLMKANMMTNSGHFNVENMIASLPAEQHAIARECAMIEQGGDLCDLA
YRNQMCMRDKSPDFYADHIKMMATAAPAPSK

>TpalOBP83a-like
MKTLLAVAAALLAVSALVTAEVNLSDDQKALLKQLRGSCMEETGVAEATIEACKTGNFAD
DPKLRCYLKCVYQQMTVMDDDGMVDADMMVTMLPEEIQAKAEPILQVCKAVNGADAC
ENAMQFNKCLYEKAPDYYIVP

>TpalOBP72-like
MEASRRDRRDGVSLLLAATAILALTWHAASASPVVTDEQLAKAAKMVRGVCQPKTGATD
AQLDALASGILGEEMEVKCYMGCLMATTRTIKNGKIDAKMMKMQAEKMMPPHRRDATI
ESLAACKDETGADNCELAFNYLTCAKKFNPEKFFFPA

>TpalOBP19d X1
MKVLVVLAACVLVCSAAAVDDAEREKAKQIKQQCKQEAGATDADVSELKDKDELTSPAA
KKMMACVFTKNKIMQNNKFSKEGSMEVARKLMAGRPGSEAKLKIAEQIAENCNKEIGSV
EASGDEMAKLIFDCLKKQAKEYLTAMNFVPREKV

>TpalOBP19d X2
MKVLVVLAACVLVCSAAAVDDAEREKAKQIKQQCKQEAGATDADVSELKDKDELTSPAA
KKMMACVFTKNKIMQNNKFSKEGSMEVARKLMAGRPGSEAKLKIAEQIAENCNKEIGSV
EASGDEMAKLIFDCLKKQAKEKIAAVKKASS

>TpalOBP19d X3
MKVLVVLAACVLVCSAAAVDDAEREKAKQIKQQCKQEAGATDADVSELKDKDELTSPAA
KKMMACVFTKNKIMQNNKFSKEGSMEVARKLMAGRPGSEAKLKIAEQIAENCNKEIGSV
EASGDEMAKLIFDCLKKQAKENGFTSPHV

>TpalOBP71
MKAVIFVAVVCLALAAVQALRCRMEEDAAGEEDYREAVRICMSNVSNDWELAGGDRHG
DRHGDRHGGNGGGGRGSHRQEQRRTYNRGPTYQSGDGDDYGDSDNRQGGQRGDRGGY
GSNSCDANSGGDNGQRGGNNMQGSSNNNNNNNNNGGDGSSRGDSGGSRKGGRGGSSS
SNRGSRPRTRRAVRRRGGDASMFDQVDACIVHCIFRQMKMVNENSHPDKSSVVSAMTQRI
RDPELKDFIQESIHECFDILMSGNIGGRCDFAKNLALCLEEKGRRHCEDWNHDMNASRQN
KADRGSGQQRPPRQHSGAYQG

>TpalOBP70
MHALTVVAVAVLAAAVADAAAPQVRGRCATPPAAPQRIEKVINECQDDIKLSIIEEALSVLG
ESATELISKEVKGRAPAKSAKSRAKRQAFTEDERRIAGCLLHCVYKKVKALNPAGVPTGEG
LVKLYAEGVQDASYFLATAQAVNHCLGAAQQQGRLLPQSVKEPGQKCYLAYDLFECVSDQ
IIEYCGASP

>FoccOBP1
MDRPAALLCLAVLVALAATALCYPRPAPVVTPEQYEKSLKMLRNVCQPKTGMPADMLER
MKGGEFVQDEKAYCYTACFLQTMMVLKNNKVDSKMFKMQVKKMMLPDAAASVINAFT
ECEGTPAGAEPCETTGLFINCVKKVDPTFNVPAWAASVAKSTMLHTPDMLLVPQDKNTPA
FLYPQFLIKASICYKTMKTTAPKNDKF

>FintOBP1
MDRPGALLSLLAVLLALSATALCHPRPRPAPLVTPEQYEKSLKMLRNVCVPKTGIPEDMLN
RMKGGEFVQDEKAYCYTACFLQTMMVLKNNKVDSKMFKLQVKKMMLPDDAARVIAAFT
SCEGTPAGAEPCETTGLFINCVKKVDPTFNVPAWTHHNAKPHRRLREREPRLFTWHH

>FoccOBP2
MSASTILSIVATVITSGRAAYNLDNPYYNSALFDQMDDFYMPKSENFAADSDPELRQKMSFS
NEICCGTYSDKNMMREDNVSRECYEEVFKSSDFLDSWNYFDTDAAKTVAQKVVCLQQCM
WKKSGTMDDRYDSLTGSLSDRYSEGSGSKEPNMNFPEVAVAKCLPSSNPDPASNWFGFGSS
DCNRAYLDFSYCVWEELHADCPSQLWDSDNKWCEETKEYLQARSQLTKS

>FintOBP2
MDAPTLIGVVIATVVASASAATYNLDNPYFNRSLFDQLDDYYMPNSENFVAESDPELRSKM
SFSNEMCCGTYTDKNMIREDNMSKECYDQVFKSPDFLDSWNYFNTDSASGVAQSVVCLEQ
CLWKKRGTMDDRYDSLTGRLSDSYSEGSGKKEPNMNFPEVAVAKCLPASNPDPAANWFGF
GSSDCNRAYLDFSYCVWEELHANCPARLWDFNNKWCEETKEYLLVRSQLTKKWSGLVKN
VSWIVRNTPMMISCQ

>FoccOBP3

MQGYTVLAAAALIAMASLAQADKKKELMPRYLKYTLECAKELNAEVGVCKEMMKEGND
NSADKYQPCKCVIACVAKKAKVMTDAGEANVEAFTAAVDEFEIKEWSDEWQRVKPLCEP
EVKGKKDCVLGYDFFTCGYDKSEIFRDVMKKFMGAMDKS

>FintOBP3
MQSSTVLLVAALVAMASLAQADKKKELMPRYIKYALECAKELNAEVGVCKEMMKEGND
NSAEKYQPCKCVIACVAKKAKVMTDGGDANVDAFTAAVDEFEIKEWSDEWQRVKPLCEP
EVKGKKDCVLGYDFFTCGYEKSEIFRDVIKKFMGAMDKS

>FoccOBP4
MKTLLTLAAALLALCGLASAGVELSEDQKQLMKQLSSAYMAETGVDEATIDACKTGQFAD
DPKLKCYLKCTYQQMTVMDDDGVVDADMLLTMLPEEIQPKAEPVVNACKEMRGADAC
DNAMMFNKCLYEKAPDYYMVV

>FintOBP4
MKTVLPLVVVLLALCGMASAGVELSEDQKQLMKQLSTACMAETGVDEATIDACKSGQFA
DDPKLKCYLKCTYQQMTVMDDDGAVDADMMLSMLPEGMQPKAEPILNACKDLRGTDA
CDNAMIFNKCLYDKAPEYYMVV

>FoccOBP5
MKVFVLLAVALVAYASADEAARERAKAIKKECKEQAGATDDDLKELKEKDELLSPVNKKL
MACVFSKNKIMQNNKFSKEGSMEVARELMSGRPESETKLKIAEQIADNCAKEIGNVETTG
DEMAKLIFNCLKKQAKEYRKAMATVPNQDISQSSW

>FintOBP5
MKVLLIALALAACAACAVADDAARERAKAIKKECKEQVGATDDDVKELKEKDQLLSPVN
KKLMACVFSKNKIMQNNKFSKEGSMQVARELMSGRPSSESKLKIAEQIADNCAQEIGDVET
SGEEMAKLIFNCLKKQAKESGFPSPHVW

>FoccOBP6
MKAVNVVVLVVAAVLAVASAEDNRDKLIPEYMAAAMDCVKDYEMDFTVCKEMMKDGV
NLAEEKFTPCKCVPACVAKKRKLMSEDGEYDVDAFTKAVNEFGYEPWSEEYKKVFPICKDS
YKGKKNCEAAAALTPCKCVPACVAKKRKLMSEDGEYDVDAFTKAVNEFGYEPWSEEYKK
VFPICKDSYKGKKNCEAAAALAVCAWKNSKMMRDTVGQYMGQMDGE

>FintOBP6
MKAVNVVVLAAAAALVQPAAAEDNRDKLIPEYMAAAMDCVKDYEMDFTVCKEMMKE
GVNLAEEKAAEDNRDKLIPEYMAAAMDCVKDYEMDFTVCKEMMKEGVNLAEEKYKPC
KCVPACVAKKRKLMSEDGEYDVDAFGKAVFEFGYEPWSEEYKKVFPICKDSYKGQKNCDA
AGAYKPCKCVPACVAKKRKLMSEDGEYDVDAFGKAVFEFGYEPWSEEYKKVFPICKDSYK
GQKNCDAAGALAVCAWKNSKMMRDTIGQYMGQMDGE

>FoccOBP7
MHILPVAVLALAALAAVEAAAPQLVRGRCATPPAAPQRIEKVINECQEDIKLSILEEALSVL

GESTGIISSDAKTRQPGKSSARRQAFTEDESRIAGCLLHCVYKKDAKTRQPGKSSARRQAFT
EDESRIAGCLLHCVYKKVKALNSAGLPTGEGLVKLYAEGVQDASYFLATAVKALNSAGLPT
GEGLVKLYAEGVQDASYFLATAQAVNHCLGAAQQQGSLLPQAVKEPGQKCYLAYDLFECV
SDQIMEYCGASP

>ApisOBP1
MLNLKVMMFLCLSVIVVYCESDQVPINSSAAVESCLLETNMTRDEFEDMLTSPNARELTILK
SHAHKCMFGCVMRKNHIVNDGVVSKEVLSKYVLNFYGRPDYKRRLIIKDVEHIVDVCAKK
VADESETDECELAATLVTCIVLEANKAGLVDDPARQI

>ApisOBP2
MKVSAATAVLVALVATVQSSDPCNISTCYKSGTTKPPMAVTPTHLPVQSSSTQTSHPQTTYA
KDHVHGSTTTKSGVNATVTTASGASVNGTEPPAVVKSSAGVTGNSTTPKPTMTEGHVALK
QKLNTIAVKCKDELHAPQEIMALVSNTVVPQNEQQRCYLECVYKNLNLIKNNKFSVEDGK
AMARIRFANQPEEHKKAVTIIETCEKEAVIDPKTTEKCAAGRVIRNCFVKNGEKINFFPKA

>ApisOBP3
MISSTFYITLVFGIAMLISCGHGRFTTEQIDYYGKACNASEDDLVVVKSYKVPTTETGKCLMK
CMITKLGLLNDDGSYNKTGMEAGLKKYWSEWSTEKIESINNKCYEEALLVSKEVVATCNYS
YTVMACLNKQLDLDKST

>ApisOBP4
MRGNYSSMVFLLFAIGFQDIFCQKQEPSGKCRAPDKAPLNLEIIINTCQEEIKSALLQEALDI
LNDGNVEQNTPNYSSRSKREAEEDLTNEERRVAGCLLQCVYKKVKAVDETGFPVVDGLMK
LYNEGVQDRNYYIATLSAVRHCISIAQQLKQQQPSKSFDDGQTCDLAYEMFECVSEKIEENC
GVENKSNN

>ApisOBP5
MSANSATIKCIAVAAILLQISVIFADAGHHRRGKELLDTEDSDFFRCKQASRKSCCGPENAM
KRFGDKDKVAADECYAQVAEKFATVTATTPKQDLFSAEAVKITKKKQFCLHECIGKKNNLL
TEDGSLNKTFIADYAMKSVFKEQWQKQVGQKALDKCLEETYIPWPAEDKENVCNPVYVQ
FQHCLWLQYESNCPANKIKITKKCEKTRNRYRMQKSTSN

>ApisOBP6
MQKVVFICIFAIICQTVFTAGYDRTWILRQKRGTNDDECRTLLPSSEKKLPSCCQMPNILPNL
DSTWEKCFETFKQFKDKPETKEYKEMAHGKEPPCLFQCIFMQSGLTTSDGKLNEDAITKKM
SEGINNDEKWKSIWQNSLNKCFDDVKQEDKKQILIMNTPAGRLMKCFLRDMYMSCPKNV
WVESSECLNMKDLVQKCPEMPPPVFKSPPKLI

>ApisOBP7
MVARKRMYNMLPTTVLFAIIAATVLKDCDAYLSEAAIKKTQQMLKTVCSKKHSVEEDVFT
NIKKGIFPEDNNNIKCYFACNFKTMQLINQKGVIDKKMFKDKMSMMAPPNVYKILLPVIE
QCTGKDKGEELCQSSYNVIKCAHSVDPKSLEFLPL

>ApisOBP8
MFALKVACLCLSVAVVFGENNQQNGPSDRSATIFQSCIAETKLSGDALKGFRSMSIPKTQAE
KCMMGCLMRKVNVINKGKFSVEEATKVAQKYYGTNEAMMKKAKDLIDVCAKKAQSTTE
ECALAGIVTTCIVEEAQKAGLSGGPGSRSRRTVSPKFRRDAMAQKAGLSGGPGSRSRRTVSP
KFRRDAM

>ApisOBP9
MIIKKTLLLSVFVLFGCLFSINKADDADAKDKELMSKLFTVVFKCFKDADWGTCGEMITTK
YDITQAKYKQCTCHMACAGEELGMINASGQPEPAKFLEYVNKINNPDIKSQLQLIYDKCQ
NVKGSEKCDLAEQFAICAFKESPALKERVSTLMEMLVKMKPKSK

>ApisOBP10
MEHLRSTNVVFAIVMALLVVQSSTRPQPDEMEEIKRTLYNACAGKFPITEEIKNNAKNSIISD
DPTFKCFLKCCFDEMSMIDEDGIIDGDSLKAMAPDHIKPILEQVIPSCTKNVKQDGCEASFE
FISCGIKLNPLIVALLPL

>ApisOBP11
MSSSTFYITLLFGIAMLISCGYGIFTTEQIDYYGKACNASEDDLIVLKSYKVPSTETGKCLMKC
MITKLGLLNDDGSYNKTGMEAGLKKYWSEWATEKIETINEKCYEEALLVSAEIIATCNYSYT
VMACLNKKLDLDKLT

>ApisOBP12
MEHLRSKNVVFAIVMALLIRPKTDGNDAIEEMKNTLYNICSTKYRITEELKNNVKKSIISDDP
TFKCFLKCCFDEMSLIDEDGIIDGDSMIQMTSDDDKLIAEQVIPNCIKTVKQDSCEAAFDFVS
CVIKLNPSTAALLPL

>ApisOBP13
MSLLLNSGGCCGHSTTTVMTLSVAILLTVGQVHNLKCRTADHSRRHGEFIDIAEQCNNTTS
GDRPASAVDGDYSTYASGGDNDGRWNGDGRRNNNNNREFSVNRQDFTGNRGSGGGYD
RDQSSCSEARPQQYSGNTGSRSKNHRQGSYPSADYDSGQSNSNYNRQSLPTRRYRRDDSNE
KSKRQKAAVTGSNNRLLGNNRFRNMTKTGGNQPGKGTYLDKMDACTIHCVFNQLEMLN
SNSRPDKYSIVNIMTNQIKDVELKEFIQDSIDECFDTLELDSHNNKCEFSKNFAVCMENKAQ
RNCDDWDENLSANKINSAGLQDGTNQQDKRKGY

>ApisOBP14
MSSSTFYLTLLFGIAILISCGYGTFTTEQIDYYGRACKASEDDLVVVKSYKVPSTETGKCLMKC
MITKLGLLNDDGSYNKTGMEAGLKKYWSEWSTEKIEAINNKCYEEALLVPKEIVATCDYSY
TVMACLNKQMDLDKLT

>AmelOBP1
MASNTKQAFIYSLALLCLHAIFVNAAPDWVPPEVFDLVAEDKARCMSEHGTTQAQIDDVD
KGNLVNEPSITCYMYCLLEAFSLVDDEANVDEDIMLGLLPDQLQERAQSVMGKCLPTSGSD
NCNKIYNLAKCVQESAPDVWFVI

>AmelOBP2
MNTLVTVTCLLAALTVVRGIDQDTVVAKYMEYLMPDIMPCADELHISEDIATNIQAAKNG
ADMSQLGCLKACVMKRIEMLKGTELYVEPVYKMIEVVHAGNADDIQLVKGIANECIENAK
GETDECNIGNKYTDCYIEKLFS

>AmelOBP3
MKTIVILLFTLCIVSYMMVRCDDITLCLKQENLNLDDIDSLLEDESERMLRKRGCIEACLFHR
LALMNDNVFDVSKFDVYLNDTDMDMDLKDSIRKIIRQCVDNAKNEDKCLTAQKFSRCVI
DYVKFHITQYMISNANSNTTSEEESSDNST

>AmelOBP4
MKITIVSLLCVIYCALVHADTVAILCSQKAGFDLSDLKSMYESNSEEQMKKLGCFEACVFQK
LHFMDGNTLNVEKLESGTRELTPDDFTEDVHEIIEQCVSKAADEDECMVARKYIDCALEK
MKFLDDELEKIAGN

>AmelOBP5
MHVKSVLLLITIVTFVALKPVKSMSADQVEKLAKNMRKSCLQKIAITEELVDGMRRGEFPD
DHDLQCYTTCIMKLLRTFKNGNFDFDMIVKQLEITMPPEEVVIGKEIVAVCRNEEYTGDDC
QKTYQYVQCHYKQNPEKFFFPPEKFFFP

>AmelOBP6
MKGLGVSLLVALLLVLLAIEDTMSKKMTIEEAKKTIKNLRKVCSKKNDTPKELLDGQFRGEF
PQDERLMCYMKCIMIATKAMKNDVILWDFFVKNARMILLEEYIPRVESVVETCKKEVTSTE
GCEVAWQFGKCIYENDKELYLAP

>AmelOBP7
MKKFLVIFVYILSVAVIIRANGINEILKIMAVSMKDIRYCIIHMGLTFKDFIKMQELLQEEDISE
GNIKKYLTNYSCFITCALEKSHIIQNDEIQLDKLVEMANRKNISIDVKMLSECINANKSTDKC
ENGLNFIICFSKLLSDMYEDTFEDTLKHKSYV

>AmelOBP8
MTIEELKKTIKNLRKVCSKKNDTPKELLDGQFRGEFPQDERLMCYMKCIMIATKAMKNDVI
LWDFFVKNARMILLEEYIPRVESVVETCKKEVTSTEGCEVAWQFGKCIYENDKELYLAP

>AmelOBP9
MFKNYHFFFILVITLIFLYFGEADIKKDCRKESKVSWAALKKMKAGDMEQDDQNLKCYLKC
FMTKHGILDKNAEVDVQKALRHLPRSMQDSTKKLFNKCKSIQNEDPCEKAYQLVKCYVEF
HPEVLQTVPFL

>AmelOBP10
MKYSILLSLLITCLICSPSVHCGTRPSFVSDEMIATAASVVNACQTQTGVATVDIEAVRNGQ
WPETRQLKCYMYCLWEQFGLVDDKRELSLNGMLTFFQRIPAYRAEVQKAISECKGIAKGD
NCEYAYRFNKCYAELSPRTYYLF

>AmelOBP11
MKAAEIWLVSLYWYLILQIALVYGEISDIDEFREMTSKYRKKCIGETKTTIEDVEATEYGEFPE
DEKLKCYFNCVLEKFNVMDKKNGKIRYNLLKKVIPEAFKEIGVEMIDSCSNVDSSDKCEKS
FMFMKCMYEVNPIAFIAP

>AmelOBP12
MLYNNLTIVIILIMCGVQNLRARSVNIFQDIADCVDRSNMTFHELKKLRDSSEARIKLINEEE
NFRNYGCFLACIWQQTGVMNGSELSTYNIAGIIEGQYHDDEDLKTFFHKIALTCEDDVHRK
FLHVNDECDVALSFKLCMLKAMRNYP

>AmelOBP13
MKTIIFIFAFCLVGILAVSEESINKLRKIESVCAEENGIDLKKADDVKKGIFDKNDEKLACYVD
CMLKKVGFVNADTTFNEEKFRERTTKLDSEQVNRLVNNCKDITESNSCKKSSKLLQCFIDN
NLMKIFE

>AmelOBP14
MKTIVLIFGFCVCVGALTIEELKTRLHTEQSVCKTETGIDQQKANDVIEGNIDVEDKKVQLY
CECILKNFNILDKNNVFKPQGIKAVMELLIDENSVKQLVSDCSTISEENPHLKASKLVQCVS
KYKTMKSVDFL

>AmelOBP15
MKTILIISAICICVGALSIKDFQNAIRMGQSICMAKTGINKQIINDVNDGKINIEDENVQLYIE
CAMKKFSFVDKDGNFNEHVSREIAKIFLNENEINQLITECSAISDTNVHLKITKIFQCITKFKT
INDILNS

>AmelOBP16
MKTFVIIFAICVCVGAMTHEELKTGIQTLQPICVGETGTSQKIIDEVYNGNVNVEDENVQSY
VECMMKKFNVVDENGNFNEKNTRDIVQAVLDDNETDQLIVECSPISDANVHIKISKIFQCF
MKYKTITDILNS

>AmelOBP17
MKTIVIISAICVCVSAMTLDELKSGLHTVQSVCMKEIGTAQQIIDDINEGKINMDDENVLLFI
ECTMKKFNVVDENANFNEKISSDIVRAVLNDNEADQLLAECSPISDPNALIKISKILECFFKY
KTINQILNS

>AmelOBP18
MKTFVIISAICVCVGALTLEEFQIGLRAVVPICRIETSIDQQKEDDFRDGNIDVEDEKVQLFSE
CLIKKFNGYDDGGNFNEVVIREIAEIFLDENGVNKLITECSAISDADLAVKSAKLLKCIGKYK
TLKEMLSGYDDGGNFNEVVIREIAEIFLDENGVNKLITECSAISDADLAVKSAKLLKCIGKY
KTLKEMLSG

>AmelOBP19
MKTIVVIFAFCICVNAMTIEELKIQLRDVQEICKAESGIDQQTVDDINEVNFDVEDEKPQRY
NECILKQFNIVDESGNFKENIVQELTSIYLDENVIKKLVAECSVISDANIYIRFNKLVKCFGKY

KTMKEVLNL

>AmelOBP20
MKTIVVIFAFCICVNAMTIEELKIQLHDVQEICKTESGIDQQTVDDINEVNFDVEDEKPQRY
NECILKQFNIVDESGNFKENIVQELTSIYLDENVIKKLVAECSVISDANIYIRFNKLVKCFGKY
KTMKEVLNL

>AmelOBP21
MKTIVIISAICVCVGALTLEELQIGLRAVIPVCRIDSGIDEKKEDDFRNGIIDVENEKVQLFSEC
LIKKFNAYDDGGNFNEVVVREIAEIYLDENEVNKLITECSAISDADIHLKSSKLIKCFAKYKT
LKEIMNE

>DmelOBP18a
RVNAEGCLKHHNLTSAQVQAVAPSTPVADVPVAVKCYSRCLIQDYFGDDGKIDLQKVGKR
GSQEDHVILSQCKQQFDGVTNLDTCDYPYLILQCYFKGKQSGTIAS

>DmelOBP19a
GVTEEQMWSAGKLMRDVCLPKYPKVSVEVADNIRNGDIPNSKDTNCYINCILEMMQAIKK
GKFQLESTLKQMDIMLPDSYKDEYRKGINLCKDSTVGLKNAPNCDPAHALLSCLKNNIKV
FVFP

>DmelOBP19b
DEEEGSMTVDEVVELIEPFGDACTPKPSRENIVEMVLNKEDAKHETKCFRHCMLEQFELMP
EDQLQYNEDKTVDMINMMFPDREDDGRRIVKTCNEELKAEQDKCEAAHGIAMCMLREM
RSSGFKIPEIKE

>DmelOBP19c
QTQAFDLAKLLPKTGTEPIWAVIDRNLPQVQELVTAARMECIQKLQLPRDQRPLGKVTNPS
EKEKCLVECVLKKIKLMDADNKLNVGQVEKLTSLVTQDNKMAIAVSSSMAQACSRGISSK
NPCEVAHLFNQCISRQLERNNVKLVW

>DmelOBP19d
KPHEEINRDHAAELANECKAETGATDEDVEQLMSHDLPERHEAKCLRACVMKKLQIMDE
SGKLNKEHAIELVKVMSKHDAEKEDAPAEVVAKCEAIETPEDHCDAAFAYEECIYEQMKE
HGLELEEH

>DmelOBP22a
TKEPEEVKIVSECAKENNVHRKKALDLLMSYRLKKKTHNVMCFINCIFERTNILQKVKEKV
VKENHNCDSIKDADKCAESFQKFQCLVKIEMKVRGIDRG

>DmelOBP28a
FDEKEALAKLMESAESCMPEVGATDADLQEMVKKQPASTYAGKCLRACVMKNIGILDAN
GKLDTEAGHEKAKQYTGNDPAKLKIALEIGDTCAAITVPDDHCEAAEAYGTCFRGEAKKH
GLL

>DmelOBP47a
RFAKININLGLTVADESPKTITEEMIRLCGDQTDISLRELNKLQREDFSDPSESVQCFTHCLYE
QMGLMHDGVFVERDLFGLLSDVSNTDYWPERQCHAIRGNNKCETAYRIHQCQQQLKQQ
QQNLLATKEVEVTTTPAGSDETKP

>DmelOBP51a
LFESEANECAKKLGITPDYFENFPHSSRVKCFYHCQMEKLEIIANGVVTPFDLKVLNISPE
SYDKYGVKVKPCLKLSHRDKCELGYLVFQCLKREFNL

>DmelOBP56a
SSLNLSDEQKDLAKQHREQCAEEVKLTEEEKAKVNAKDFNNPTENIKCFANCFFEKVGTL
KDGELQESVVLEKLGALIGEEKTKAALEKCRTIKGENKCDTASKLYDCFESFKPAPEAKA

>DmelOBP56b
QSAAELAAYKQIQQACIKELNIAASDANLLTTDKEVANPSESVKCYHSCVYKKLGLLGDDG
KPNTDKIVKLAQVKLAQIRFSSLPVDKLKSLLTSCGTTKSAATCDFVYNYEKCVVKGISAIRF
SSLPVDKLKSLLTSCGTTKSAATCDFVYNYEKCVVKGISA

>DmelOBP56c
KAWVMFFIFYISFTRSLSVSLNMSMTRTLVPDPPNGTENKLSQEMLRACMRRTEISMSQLKL
FHMSLMNSDYNNDNDIAPTPVQSIGDVNNLGDLDFNGNSQMPYLDLKHNEPLQCFVSCL
YETLDLDRYNVLLEEAFKNQVQTIIQHEKAEIKECSDLQGKTRCEAAYKLHLCYNHLKTLE
AEQRIREILERTEAENEGFGPEGSDFIDGIQHSGEAMTTAKSE

>DmelOBP56d
ELQLSDEQKAVAHANGALCAQQEGITKDQAIALRNGNFDDSDPKVKCFANCFLEKIGFLI
NGEVQPDVVLAKLGPLAGEDAVKAVQAKCDATKGADKCDTAYQLFECYYKNRAHI

>DmelOBP56e
SAVGLTDSQKAEAKQRAKACVKQEGITKEQAIALRSGNFADSDPKVKCFANCFLEQTGLVA
NGQIKPDVVLAKLGPIAGEANVKEVQAKCDSTKGADKCDTSYLLYKCYYENHAQF

>DmelOBP56f
MKSSEKIKACLKRQLGYTITENTKFDAKEDSLQSKCFYHCLLEVKGVIANDAISSEQPRKVL
EKKYGITDTDELEKAEEKCHSIKASGKCELGYEILKCYQSITKH

>DmelOBP56g
QQANIDSSVSKELVTDCLKENGVTPQDLADLQSGKVKAEDAKDNVKCSSQCILVKSGFMD
STGKLLTDKIKSYYANSNFKDVIEKDLDRCSAVKGANA CDTAFKILSCFQAAN

>DmelOBP56h
NPDFRQIMQQCMETNQVTEADLKEFMASGMQSSAKENLKCYTKCLMEKQGHLTNGQFN
AQAMLDTLKNVPQIKDKMDEISSGVNACKDIKGTNDCDTAFKVTMCLKEHKAIPGHH

>DmelOBP56i
GPIKDQCMAAAGITAQDVANRHETDDPGHSVKCFFRCFLENIGIIADNQIIPGAFDRVLGHI
VTAEAVERMEATCNMIKSETSHDESCEFAWQISECYEGVRLSDVKKGQRTRNHRG

>DmelOBP57a
KESQPFDFFEGTYDDFIDCLRINNITIEEYEKFDDTDNLDNVLKENVELKHKCNIKCQLERE
PTKWLNARGEVDLKSMKATSETAVSISKCMEKAPQETCAYVYKLVICAFKSGHSVIKFDSYE
QIQEETAGLIAEQQADLFDYDTIDL

>DmelOBP57b
RHPFDIFHWNWQDFQECLQVNNITIGEYEKYARHETLDYLLNEKVDLRYKCNIKCQLERD
STKWLNAQGRMDLDLMNTTDKASKSITKCMEKAPEELCAYSFRLVMCAFKAGHPVIDSE

>DmelOBP57c
QSLSLLEETNYVSDCLASNNISQAEFQELIDRNSSEEDDLENTDRRYKCFIHCLAEKGNLLD
TNGYLDVDKIDQIEPVSDELREILYDCKKIYDEEEDHCEYAFKMVTCLTESFEQSDEVTEAGK
NTNKLNE

>DmelOBP57d
MPEKMSLRLVPHLACIIFILEIQFRIADSNDPCPHNQGIDEDIAESILGDWPANVDLTSVKRS
HKCYVTCILQYYNIVTASGEIFLDKYYDTGVIDELAVAPKINRCRYEFRMETDYCSRIFAIFNC
LRQEILTKS

>DmelOBP57e
NPCVSQNELSEYEAHQVMENWPVPPIDRAYKCFLTCVLLDLGLIDERGNVQIDKYMKSGV
VDWQWVAIELVTCRIEFSDERDLCELSYGIFNCFKDVKLAAEKYVSISNAK

>DmelOBP59a
LKCRSQEGLSEAELKRTVRNCMHRQDEDEDRGRGGQGRQGNGYEYGYGMDHDQEEQ
DRNPGNRGGYGNRRQRGLRQSDGRNHTSNDGGQCVAQCFFEEMNMVDGNGMPDRRK
VSYLLTKDLRDRELRNFFTDTVQQCFRYLESNGRGRHHKCSAARELVKCMSEYAKAQCED
WEEHGNMLFN

>DmelOBP69a
VEINPTIIKQVRKLRMRCLNQTGASVDVIDKSVKNRILPTDPEIKCFLYCMFDMFGLIDSQNI
MHLEALLEVLPEEIHKTINGLVSSCGTQKGKDGCDTAYETVKCYIAVNGKFIWE EIIVLLG

>DmelOBP76a
MTMEQFLTSLDMIRSGCAPKFKLKTEDLDRLRVGDFNFPPSQDLMCYTKCVSLMAGTVNK
KGEFNAPKALAQLPHLVPPEMMEMSRKSVEACRDTHKQFKESCERVYQTAKCFSENADG
QFMWP

>DmelOBP83b

QEPRRDGEWPPPAILKLGKHFHDICAPKTGVTDEAIKEFSDGQIHEDEALKCYMNCLFHEF
EVVDDNGDVHMEKVLNAIPGEKLRNIMMEASKGCIHPEGDTLCHKAWWFHQCWKKAD
PVHYFLV

>DmelOBP83g
KFLLKDHADAEKAFEECREDYYVPDDIYEKYLNYEFPAHRRTSCFVKCFLEKLELFSEKKGF
DERAMIAQFTSKSSKDLSTVQHGLEKCIDHNEAESDVCTWANRVFSCWLPINRHVVRKVF
A

>DmelOBP84a
LQDHAKDNGDIFIINYDSFDGDVDDISTTTSAPREADYVDFDEVNRNCNASFITSMTNVLQ
FNNTGDLPDDKDKVTSMCYFHCFFEKSGLMTDYKLNTDLVRKYVWPATGDSVEACEAEG
KDETNACMRGYAIVKCVFTRALTDARNKPTV

>DmelOBP99a
ADYVVKNRHDMLAYRDECVKELAVPVDLVEKYQKWEYPNDAKTQCYIKCVFTKWGLFD
VQSGFNVENIHQQLVGNHADHNEAFHASLAACVDKNEQGSNACEWAYRGATCLLKENL
AQIQKSLAPKA

>DmelOBP99b
DHHHHHHDYVVKTHEDLTNYRTQCVEKVHASEELVEKYKKWQYPDDAVTHCYLECIFQ
KFGFYDTEHGFDVHKIHIQLAGPGVEVHESDEVHQKIAHCAETHSKEGDSCSKAYHAGMC
FMNSNLQLVQHSVKV

>LmigOBP1
MWARLNDCAALLLLLAAAARAWDVNMKLTGRIMDAAKEVDHTCRSSTGVPRDMLHRY
AEGQTVDDDDFKCYLKCIMVEFNSLSDDGVFVLEEELENVPPEIKEEGHRVVHSCKHINHD
EACETAYQIHQCYKQSDPELYSLVVRA FDATIGD

>LmigOBP2
EMTPEFMEIVNKCKTEHEPTEDELKGMMALKVPESANGKCFMGCVLQEIGVVKDGKFDK
EEAKKHAASKMTDKDELEKHMQLIEKCSQEVGGETDSCGIGPKLMECIKQFAPEFDIALPQ
QPSE

>LmigOBP3
DAEKMKEAVDKCKASENLDSLDGLKSSKSPSTEEEKCFIGCLMMDMKLLSSDGQYDAAST
KDMINNCEYLKDKPDEKSVALEVADDCAGKATGCSGHCECGPKAVGCLIKGMVDKGYEE
SFARIDKMLEKLDD

>LmigOBP4
MGTAVAAAVLLLVAVANAEDSLMEIVIREVKGCMDSEHLNSIADLRSYNEAKSPEEKCFLG
CMLKKFKALDADGQYDAEGLKATIQHCPRMKAHPNIQQAALQVADECAGKVTGCSDYC
TCAPLATRCLHEGMKNKSFQTIFIALDEALDKMQS

>LmigOBP5
MALAARVFSATLMLTAVLFGDISTAGEVFTMSQLKAAVNECNDTYFLSQKNWDTVFTTGSL
EDENDLVAKCFFECVLEKTGAMDEKGNINSDITKAVFLASHEGTGTAVQGHDDLIDMCVP
GRDETDICERGYALVKCVTVEELSRRQARK

>LmigOBP6
QLLLVSLALCLSAAVAEKQAPWCPTTASQGVQEDMGQCAEEIKDAILREYAKTVSSRRTRS
AEMSEEDRLLVGCMVSCLFRKGPHSRLQTGSKLLLAELGAMRLFSDGADDARYRNATATA
VRRCSASSRSLLPDDGGPRHECELGFFMFECVSDQITEYCQWQPE

>LmigOBP7
AAETKVMEGIKACMASEHLGSLGQLKANNEARTPEEKCFVGCVMKHLHVLNSEGQYDL
ALVKERANNCPELAKDPQKKADTLRVAEDCAAKVIGCSGYCECGVAAGECLAQGMEAK
GHETIYDFLRKIVDKMDV

>LmigOBP8
AVRLLLLLPPLLLLALSCVTAAPSITSTEMRMDMMVIQHCNETHPVALIDMNKALINKKIEP
QNTVFKCFVFCLLNKYEWMDDEGGFLIANMKHNLSDSHLDQLSIDFIVYKCSATGSSDKCE
RAYRFTECFWGEVTKFPENSDEKYEDPDLFALYQ

>LmigOBP9
TDATATAAMDKASSAAVTACLLIAVAALHTQALSLEQLRQTSKIVRNMCLKKTGVDLALVE
GIQEGQFPDNQDLKCYMKCCMGAMQVLRQGRYNVNAAKNQADKMLPPDLKGRFIDML
DACSDRGDGVDDDCEMAYQLTKCSYETDKEIFLFP

>LmigOBP10
AISESMSRAEEAASKIDIPELFEECNETFTIPKVTLNYFFSHGRLQNENDYGSKCFVHCLTDRS
GEIDSDGNFDVDLIKVMTRRFPNETNIEGLNEMVETCVADRGETDFCERAYGLVSCLVKEK
LARLGNSH

>LmigOBP11
VATMRSLLPVAVSAVLLVAPSKTLEPDFTKGISDVKACMASENLDSLDALRTNKEARTAEEK
CFIGCIMKFVEVLNSDGQYDVALFKDHINGCPEMAKDQQKKAALLEVAESCAGKASACSG
HCECGVIVANCL

>LmigOBP12
AVILTAASTLWFAAAAFAAMVTTEIPTEDILQRVQVCNKTYPVSQEMLRSLASTGGLLSDES
DVNTRCYLECYERLGGTVNKDGKFNPEKAVTLLVSYYPKIAELGVDSVTEILKNCNSKSGT
GQCMTSYLIRNCFIAGLNAKSPHTSVFDTSSSHI

>LmigOBP13
CDPRHDTATMSALFTCCVAAWLLLAAALLQPTKGDDVWHNTDIPATMAECNATFRLGW
RCWDNLLSDGHVIDESKYQQKCWFYCLLDETGSMHADGAFDKDLLKTVLQGFPNGSSLA
HLDETTYTCVAQRNEVDLCERAYAVVKCIMTEELSRMHQSS

>LmigOBP14
MCLVVLLFGFVQSLSLLFVPDSCLLLFVVVMFYFPCKISQANGSFVMTMLCLEQTCKMMDQ
LHQTCVGESGVSEGNIDAARKGNFIDDGNLKCYMKCIFVQMTCMSDDGVFDADTAIAML
PDNLKDVASKALNACKGEKGSDACDTAFKINQCLFKQAPKDYILV

>LmigOBP15
MFYFYAFTFCCLLWMLFHCSVNCVNVDIETIWRECNETFPASEEALISFGKNGTIPDENDSV
ARCFTDCYGKKTTLLTSDGSLNWTTLDFLMRSYDMKPTAKETFGKCQKNTSNVECMKSYL
SLRCVAETVESLTDIR

>LmigOBP16
MNWGLWLPVTIALVLQLSISEALKCHTDEDTQNPDEFQEVAAMCMKNTSGSELNRNDRES
KRNGNNYHKTNFENTNDNWNSGGMGQTFPGYNSENEGYGFRGSGRCNANGDGYNGN
RNNMNQNNMNGMRQRPRNRNRRSGQQSETADVDLEDIEPCAVHCIFRQMGMLGDDAI
PDRSAVAKVMLRGVKDTEVKDFVQEAVEDCFDQVESDRKGSKCEFSKNVALCLRQKGREN
CEDWGEQDGDQQSNQNKNGNNNGNYSNNSNQYGNKKWN

>LmigOBP17
MKAFQICTLICAVVAHCMCDKEEAMKILRASVDKCSAGYGLSRETTQYIVRHNFTIKDEND
ENQRCFVQCVGQEMGDFNSEGIFDVDHATETAEKWLEWNGRTKSNLREEMEECAKITGT
GTCMTTYLITKCAMKAGE

>SexiGOBP1
MLFLLRALPLLAAVLPLRADVNVMKDVTLGFGQALDKCRQESQLTEEKMEEFFHFWRDDF
KFEHRELGCAIQCMSRHYNLLTDSSRMHHDNTEQFIKSFPNGEVLARQMVELIHSCEKQY
DHEDDHCWRILHVADCFKQGCVQRGIAPSMEMMMTEFIMEAEAR

>SexiGOBP2
MTSKCCLLLVAMATITAEVMGTAEVMSHVTAHFGKALEECREESGLSAEVLEEFQHFWRE
DFEVVHRELGCAIICMSNKFSLLQDDTRMHHVNMHDYVKGFPNGHVLSEKLVELIHNCE
KQFDSMTDDCERVVKVAACFKVDAKAAGIAPEVAMIEAVMEKY

>SexiPBP1
MAFCRSATMSVRVALVVAASMLVVVQASQDVMKNLAINFAKPLDDCKKEMDLPDSVTTD
FYNFWKEGYELTNRQTGCAILCLSSKLEILDQELNLHHGRAQEFAMKHGADETMAKQIVD
MIHTCAQSTPDVAADPCMKTLNVAKCFKLKIHELNWAPSMELIVGEVLAEV

>SexiPBP2
MAGAKWWFVCVVFALYLTSAALGSQELMMKMTKGFTKVVDDCKAELNAGEHIMQDMY
NYWREDYQLINRDLGCMILCMAKKLDLMEDQKMHHGKTEEFAKSHGADDEVAKKLVSII
HECEQQHAGIADDCMRVLEISKCFRTKIHELKWAPNMEVIMEEVMTAV

>SexiPBP3

MGSHNVFVALVLLAVGMRVAEPSKDAMKYITSGFVKVLEECKQELNMNDHIIADLFHFWK
LEYALLSRDTGCVIICMSKKLDLLDANGRMHHGNAQEFAKRHGAGDDVASKIVQIIHDCE
KKHERDDDECLRVLEVAKCFRTGIHDLDWQPKVEVIVSEVLTEI

>SexiPBP4
NFWKEGYEFTNRQTGCAILCLSSKLELLDQEMKLHHGKAQEFAKKHGADDAMAKQLVD
LIHGCSRSTPDVTDDPCMKALNVAKCFKAKIHELNWAPSMDLVVGEVLAEV

>SexiOBP1
MKRSLCALVVVSAVLKLASGYYEEPSYTRDKRSPPREGKDLGIFHPYQDLIPRHCWSRPKNV
DMYKCCPIPQLYSDEVLDICGIEKIKEDEESPTKRSPTKLACKDGMCLMKESNLLDKDDRVD
YEKLRAYLDQWAEEHPNFTEAILEAKKQCAYPDGQEKDKAECEPDQIFVCLTAFIIWHCKFI
DEPECKELQAHVDECRPYYQKPKEQDKKMAKKGKKFRS

>SexiOBP2
MITSSSILVLAALVQVLFAQQPDFQSGPPGPPGPHHKGPPPGAFPPGIPKSCWVPPREVNLF
KCCPIPPLYSDEVMQSCGFEKPSEDGPKPPKRHRRPDGTCKEGYCVMGNADLLQTNNSVD
YEKFRSYLDNWAASNPDFAEAIQIAKEDCAQDGGPAGPPVCEPDRLFFCLTSKIFWNCKLR
DEDGCQALQQHMDECRQYYTKPKEQIEGNAERR

>SexiOBP3
MLSMKTVIALVGLLLYEAGSVLGKNEDSVLRVISSSIGDTVLECQGEMDFKDEVIHNFMNF
WNRTNSLGTKDLGCALVCVFEKNAFLSPDGNTVLSNNVRQFLRASGADELMSLRTLDLFE
MCKNEVKQVINRCDNALELGKCFRYGIFQLHWNPEPHYWTRKEEPSKIPKQVTELQKAPR
PLSRKPVPAWRRRTDLRRIVGYVNRNCH

>SexiOBP4
MLKLCVFLALSFVACHGAPNKASGTFCGVTPNNMFKCLNNPRVLNLEAAAKCTSQVTEC
EKITCVFREQKWSKRGVIDKAKIRAHFEQYETEHPEWAQAVQHVKAFCLAPELRAQGVFL
NCPAYDIMQCALASFIKHASPSVWSTEQNCDYPKAYAADCPVCPSDCYSAAIPIGSCNACY
LQPRTV

>SexiOBP5
MFSHILHHALKLHMFYLFYLNKSSFFLSKLFLSYLTLSISFQSLSDREKSAIQKELTSVGLQCIQ
QHPLSLSDIRSFRNKMIPDGKKPKCFVACLFKKIGVMDDMGMISPMKAQENAKKVFKDNE
EYIKNVNEIMEKCSSVNQQNTSDGNKGCDRAKLAFNCFTENADKVSYLF

>SexiOBP6
MKEGNRYSHERRITNDSGDQLMVINATDDDYSGYGSGNMGEKLLTSVPRPASSNNNINKN
NTRRTRRNEPLLNRPDSDQCLSQCVFANLQVVDSRGIPREAELWNKVQTSVTSQQSRSALH
DQIRACFQELQSEAEDNGCSYSNKLERCLMLRFSDRKVDGKGNPKKSSTEQT

>SexiOBP7
MTRVLLAIGLTVITFALTQSANTKTSAMPKEAMTTTTMSDQDSSVDNNVDVDVIAVMNAC

NESYRIEMAYLESLNESGSFIDENDKTPKCFIRCVFENVGIVSEDGMQLNPARAAVIFAGQR
NGKPMDDIGDMTALCAADRQETCPCDRAYKFIRCLMSMEI ERYEKS

>SexiOBP8
MSKFTFLVLCVVAVSLSKVYASDEDKAKLHEALKPLVEECMKEHEVSLDDLKAAKEAQSA
DGVKPCFLACVYKKAEVLNSKGEFDADHALDKLKEFVSDEDVLAKVAEVGNTCKAVNDK
AVGDGDAGCERAALLTACFLEHKAEVKPIRPLLFPWGHHHHH

>SexiOBP9
MARRQRGAMFTETLPLFVILVAVTHGGKDKPVFSDEIKEIIQTVHDECVAKTGVAEEDITNC
ENGIFKEDAKLKCYMFCLLEEASLVDDDDTVDYDMLVSLIPDEYYERTTKMIFACKHLDTP
DKDRCQRAFEVHKCSYEKDPDLYFLF

>SexiOBP10
MSRFGVLSFVVLIFCMGNIYALSPEEELSIKEALHPFVVECAEEYGMTEEMFEEAKKKGSAE
DIDPCFMSCFLKKTGFFDDAGKFDAEKSISFAKEHITSESAIKFLVAGAGECVQINDEDVSDG
DKGCDRAKLLFDCLTDLKKKLSE

>SexiOBP11
MCKFSVVFLYSAVMAVNIWSASCLSEEDKAAIITAIAPLAQNCGSECGLDNDDFEKYKEDG
SDMDPCFKACLMTQMGVLDKEGKYDGKGLHKAMEEADYPGDKDDAQKFLDELDRCFD
AKGDNSGSDEEAKMKRADVLFQCMQDMKEN

>SexiOBP12
MFRFLVVACVAVVAVNGLSQELKEKFMERLETVGGECAAEVGANEDDIAELIAHKMPSRH
EGECMIFCFYKHFDMMHADGSLHAEGAIKMMEPLKADDPDLYEKLMTIGKACAEEVSSL
DDKCKYATLLAQCGVKKGKEMGLDESLFE

>SexiOBP13
MSKFTCFVLCVLAVSLAEVRSNALEKAAIRAALYPLIVDCAKEHSVTLEQLKAAKAAHSAQ
GINPCFQSCVYKKTGIFNDNGEYDIANAKTKLQKFVTDEDEYARIAEVGKTCASVNDKSVS
DGAAGCERAALLTACFLEHRAQIII

>SexiOBP14
MLGSLLFVFAFSVFSLSAEALLIDDLKQKYVDSILQCSQQYPLDRADAELLQNKVMPDKEST
KCLFACVYKVTGVMSDQGELSVEGVNALSQKYLADDPEKLKKSEEFTEACRTVNDAPVSD
GARGCDRAALIFKCTIEKSPDFSFV

>SexiOBP15
MSKFTCLLLCVVAVSLSKVHATEEEKEAIRAAVRPIMEACGKEHGVTLDDLKAAKAAHSA
DGIKPCFQSCVYKKAGIFNDNGEYDIANAKTKLQKFVTNDEEYARIAAVGKTCASVNDKP
VTDGAAGCDRAALLTACFLEHRAQIII

>SexiOBP16

MSKFTCLVLCIVAASLSQAYASEEEKAAFREAIKPIVEECSKEHGVSHDELKSAKDNQNADN
IKPCFLGCVYKKAEVFNSKGEYDVDKALEKLKKFVSNDEAYAKFAEVGKKCASVNDKAVS
DGDAGCERGALLTACFLEHKAEVPL

>SexiOBP17
MSVVRYSSFVMALFCLVSVNAMSGDEEAGVRDALRPYVQECADEYGITEEQFEEAKKKAS
ADDIDPCFMSCFLKKAEFFDAQGKFDVDSTMAFAKEHLSSEPAMKFVEAVGDECVKINDE
DVSDGDKGCDRAKLLFDCIAETKKKMD

>SexiOBP18
MSKFTCLVLCVVAVSLSGVHATAEEKAAFIEAVKPYIQECSKEHGVTPEDIKSAKEAGNADG
INACFLRCVYNKAGVINDKGEYDADKALEKLKKFVSNEDDYAKFAEIGKKCASVNEKSVS
DGEAGCERAALLTSCFLEHKSEVHA

>SexiOBP19
MTSSSVVWCAVICISTVFAWYDEPYNKKGFDECIEKFHVQPREKGSFQKPDISSVDPCFWAC
GFKILGFLDSEGQYDLETTHSHYKKENLSYLGEKQEKVEEIIEQCDAALEKITGTDPKAECD
RGFQLAKCYVEDMRKLLFEDSRK

>SexiOBP20
KFLFLVACALVAVNAVSEQLKNEFIEKMTNIGGQCAKEVGANEEDIAELLAHKAPSRHEGE
CMIFCFHKHLGLMNEDGTFSKEGGLKALEPVKADDPKLYEKLVSIGKMCQEEVAKDDDKC
KYATQLTVCGVKKGKEMGLDASMI

>SexiOBP21
MSYINYFLFSVVLFVICNNSFVYSMTREQIKNSGKLIKKTCSAKNDLTEDEVKDVDKGKFIEK
KDFMCYIACVYKMGQSVKGSTINHDMMLRQVDMMFPNDMKAPVKAAIEHCRPVAKNY
KDLCEASYWTAKCIYDFDPANFMFP

>SexiOBP22
MKTYTFRLFCYIFCISLFLGQSYGMTRQQLKNSGKLMKKSCMPKNDVTEEEVGDIEKGKFIE
NRNVMCYIACVYTMSQVVKNNKLSYEAVIKQVDVMFPAEMRDAVKAAASYCKDTTKKY
KDLCESSYWTAKCMYDYDAENFVFP

>SexiOBP23
MMDRKRLCFLIIAMYLAQGSDAMSRQQLKNSGKMLKKNCMSKNQVTEDQIGSIEKGKFV
EDKKVMCYIACIFEMTNVIKNNKLNYDSSIKQIDLMYPPELKESAKAAAEKCKDVQKKYK
DICEASYWTAKCLYDFKPEDFIFA

>SexiOBP24
MQINQLLGLLVIATCVGISHGMSRAQVKKTMSLVKNQCMPKNSVTEDQVGKIEEGVFLED
RNVMCYVACIYKNLQVVKNDKLDMGLITKQIDALYPPELKEPVKKAVSLCIHSQDNYNDL
CEKVFHASKCLYEKDPASFIFP

>SexiOBP25
PTFTYLLNTELQARTDQEIKAWFFREGMDCNNEHPLSPKEMLELKENKIPDTNSAKCFVAC
VFKKTGMLDSKGMFDAESSIAMTQKDFVDDPKKLESSKKLLEACKKVNDEAVSDGEKGCD
RSVLLHKCFVETAPQLGIKLP

>SexiOBP26
MKVFVLLVLSAYILIENEAAMTDAQLKAALKLLRNVCQPKNKATNEQIEAMHKGDWNQ
DKNGMCYMHCVLNMYKLIKKDNTLDYEVGMSTIEAQAPDSIKATAIHSLNSCKDAAKTTS
DKCIAAFEIAHCLYLDNPPAYFLP

>SexiOBP27
MLLTKIIKFLILVATCEAMTMKQIRNTGKMMRKTCQPKNNVEDEKIDPIAEGVFIDEKEVKC
YMACIMKMANTIKNGKLNYDAAIKQADLLLPDDIKEPAKEAITACKKVADAHKDICDASF
HITKCIYNHNPGIFYFP

>SexiOBP28
MKTFVVLAACVMLAQASGLTDEQKEKLKKHRSECLTETKVDEQLVNKLKGGDYKTESEPL
KKYALCMMMKSELMTKDGKFKKDVALAKVPNAADKPTVEKLIDACLANKGNTPHQTA
WNYVKCYHEKDPKHAIFL

>SexiOBP29
MFFVQAISYSTILVLFMVTSHVRTDSSEEEEKLMVKCMEEASVTKEEVKVFRTDKISDKILCF
MKCRFESEGMFDENGVIIKEMLQEGYDDFGWNDEQKIKADECIDNMKPAKECGDLADFF
SCLPVINYGELIK

>SexiOBP30
MNYAIFFFLVAILSNASGMDDDMQELINNLHNTCVGEVGVDEALITKAQNGDFAEDEKLM
CYSKCLLDQMAIVDENGIVDPEAAVAVLPADMQAEAGPAVRKCSKLRGSSPCSNVFEVMK
CWYTESPATYFLP

>SexiOBP31
MKFLVVAACVLLTVQALTDEQREKLKEHSTACAKSTGVDPEAIANAKKGTFSDDEKFKDY
LFCVSKKIGFQNEAGEIQKDVVKQKATVALKDEKLVDEIIKKCAVVKDTPQNTAFEVAKCY
YENNAKHSSLV

>SexiOBP32
MKSLVVFCIVLVVGVCANEKGNKLASECIKETGVKNELLEEAKKGIISEDPAFKAFTYCFFK
KIGIVGEDGILNRDVAIAKLPSGVDKSEAEKLLDSCKSKTGKDAVDTVFEIFKCYQQGTKSHI
MFAS

>SexiOBP33
FTCIILCVVAASLTKVSHAVTEEEKEAFREAMAPIIAECSEEHGVSEADIKAAKEAASADGIK
PCFLGCVMKKIEVLDSKGLYDAETGLGKLKKFVKDEDEFAKFEDIAKKCLKVNDESVSDGE
AGCD

>SexiOBP34
IAETKKKCLSDEDKKILRDGLDSIAHDCLHGCGIDEKELDNLDANDSIECFKKCFMTDAGF
LDLNGKYNKDVLSESLSKVTGNQDNAERILNELDRCFTENGDNSEANEEAFMKRIDILFAC
MREIRE

>SexiOBP35
SLKVLSVFVALIVALHADDDHEKHFAMFKECAEENGLKMDGFKRGERPVGPPSNEMMCT
VKCTMEKEGILSGGKILIEEFKKDPKLTKHVPADKMDAALECLKGVEVSDCSDMKKVMDC
THDMKF

>SexiOBP36
EHTMPAQRRRDKREVPFTHDEKRIAGCLLQCVYRKVKAVDGYGFPTLEGLVGLYSDGVNE
RGYFMAVLEASRECLMKNHDKFSRTVPMDNGRNCDVSFDIFECISDRIGEYCGTSGL

>SexiOBP37
QKAQIHAHFESVGMECNKGSNMITADDIASLRAKKIPAGPNAPCFLACLLKHIGIMDDSGL
LQKETALEMAKSVFQDPEELKQIEDYLHSCSGVNAESVSDGAAGCERAMLAYKCMT

>SexiOBP38
MAELARMVRENCAGETGVDVALVEQVNAGAELMPDDKLKCYIKCTMETAGMMADGEV
DIEAVLALLPPSLAEHNAPALKACGTQRGADHCDTAFRTQQCWQNANRADYFLI

>SexiOBP39
DKIAIMSAMKPIVDECAKKHGVTLEALLAAKASGKIDGIEPCFYSCVYKKTEFLNSKGEYDV
DNSLVKLKKYISSDDDYAKFSQIGKDCA

>TcasOBP1
MILKASIFLILAVATFGAILEDSELMKVVENCVKKTNANESEFSSPNFLETTPSQPALCTAKCL
LESLEIVNSEGNINMETLKEYAQPFESPAREAVATCGEEIKSVTTCDDMEKYRKCVEPLIKNS

>TcasOBP2
MNPITSVILTFLFVFSFGEKESEEALQQIFTELDGPAAELRDQCLEKNSMKVTDLKTYNTSND
IPEKELCFYKCFYEGVEFIDANGNLNVNNMKEIPAISELGDEVLNEITACVEKIGKIRCCGDL
RKIEQCYQNITM

>TcasOBP3
MWSFVTLLFSFLVLASAQKKGKYWTTISECLTEHSMGVEDMKKFDLPAEKMSEEMLCFNK
CFYDKLLITDENGEINTDNLMSIPLVNAIDASKHDDLVTCLKKVGKIEECDGVKKIEQCFVE
FI

>TcasOBP4
MCRLFVVLSLFVASQALDVEKIRNELMADKNFVELRNKCLDKLGLKEEDLRDLKFDGDVS
EDLMCFGKCIQEEDGLLDSEGNLNEEKLEKKIETMPFLSRVSDDTKNNIMECLKEIGKIETC

QDFGKQRDCIHKYV

>TcasOBP5
MAKKQLVLFFLAFIFLQSSWAYFFMSQKFAEVREECLSENSMTMDELHEGWKMENLPESHL
CFLKCLLEKREVIDENGVPQKEKIDEILTVKQLSDEKREEISTCITNVEKIENCETMSEIMRCF
PKKRRD

>TcasOBP6
MSPLLLIFISCLFPRVFGISEEMQELANTLHATCVDETGVSEDAIESARKGNFAPDDKLKCYM
KCIMEQMACIDDEGIIDVEATIAVLPEEYQAKAEPIVRKCGTKIGANACDNAFLTNKCWYE
EDPEDYFLV

>TcasOBP7
MYKTRVIYVLFALCLVEIFAIEMDDDMKELINNLHNTCTGETGATDDQIENARKGNFAEDD
SFKCYFKCVFDQMGCMTDDGKVDSEAVIAVMPPELADKIASTVRGCTEVGANPCETAWLA
NKCYQKSNPDMYFVP

>TcasOBP8
MIRYYIVLLLYFFAPPVLGISEEMQELVNQLHSTCVAETGVSEDLINKVNSDKVMIDDEKLKC
YIKCLLTETGCISDDGVVDVEATIALLPEDMKAKTTPVIRSCGAKMGANPCESAWLTHKCY
LETSPADYVLI

>TcasOBP9
MKAILLLLVATLSFYHVYCAMSEAQMKAALKLVRNVCQPKTKATNEQIEAMHTGNWDLD
KNGKCYMWCILNMYKLIGKDNSFDWEAGIATLKAQAPESVRDPAIASVNNCKDAVKTTS
DKCEAAYEIAHCMYLDNPEKYFLP

>TcasOBP10
MKTVAVLLFLALAACTKQEDDDRQETIRQYRDDCIAETKVDPALIDRADNGDFTDDAKLQ
CFSKCFYQKAGFVSETGDLLFDVIKDKIPKEANREKALAIIDKCKELKGADSCETVYLVHKC
YFLHSYGTDKKTE

>TcasOBP11
MSFLILLICVIPAIFCRSFSHDELDTDLSFIKTCNRTSPISMRTMNEVLINKKLGHGESSAFKCF
LHCLFMKYGWMDSDGGFLLHDIKQTLEESDVEIASLEFILYKCTATESNNRCERAFVFTQCF
WDKMAEQQPSEDQFFYNIEDKK

>TcasOBP12
MKLLITLATLVVATYAIDKEFVQELRQKLRSHVEACAKEVNAGPDDVSAIFAHKLPATHEG
KCIFFCMHKLYNAQNEDGSLNMAGALANLELIKDMDPDVYTKVSTSFKNCESAPFDSDPC
LYAANLVTCIVKEGRAVGLDEVLVE

>TcasOBP13
MKFLLVFLSVAILCTFAMDESFLQQTRDRVKAIVKECVTEEKATDSDFDDIMALKIPTSHEGK

CVFFCSHKKFNMQHPDGSINKEGALDTFEVVKDVDAEFHDKVITVYNHCLSTPVDPDPCV
YSVNLFQCFMKEAKAAGIHELIIK

>TcasOBP14
MNSVLFLLVCALVACSGELDKEFLMQFLQKIKKVSEDCIAETQATKNDIKTLLEHKIPDSHE
GKCMIFCFHKHFQIQNEDGSLNKVAAISLLEPIKDHSQDIYDKVVKIFNTCFDSAERDDDSC
IYASNLAECAIRESKSLGLDDLLVIE

>TcasOBP15
MNCFVIFALSLSATVFGQSLSEDEMRENARKLMTSCKDKVGASDADVEALKMHQMPESRE
GFCMLECVFDSAKIMQDGKFSKSGMIEGFKPLIGDDKAKLESLEKLSATCESELGDGEDKCE
TAKRLVECVIKNGKTHGFEVPPPRE

>TcasOBP16
MQLLVVVLAVCVLGANAGLDPKFLEKLTQEVQAVGTSCGEKEHATADDMIEIMEEKFPPTS
HEAKCVVACFYKHYKMMKEDGTFDKDAAVKAFDEIKAQDAEIHAKILKVIDACDAKKQ
MSDDHCVSAASMAGCVKTEAIANGLTKEAFMAS

>TcasOBP17
MKSTWFFLLLACSLTCALDQEFVDEFLEKMQEFGAQCAEETDATSDDIAELIARKLPPSTHE
GKCMIFCMQKKFNMMKENGGIDRAGAIAALKPLQKADPELHQKVLKIFVTCGMRVKPSP
DPCDTATELALCGKKEAEAIGLEDALLT

>TcasOBP18
MKLFILAGILFTGVCAVDQEFVEKFLQKMEKIGEECAEETHATSDDIADLIEQRDPKTHEGK
CLIFCYHKKFNTMKEDGSLDKVGSVLALEEVRDADFELYKNILTIFVTCGDKAKIYDDPCET
ATALTMCGRDEAKALGLQDAIFG

>TcasOBP19
MKYFVVFASLFLATNALSQDFIDKFVAKVKSIGETCVPETNASKDDISSLLAHKMPDSHEGK
CLIFCFHKQFQIQNDDGSINREGAIKALEPLKADDAELYEKVISIFKKCESTPVDGDSCLYAA
SLAECAVKEGRAMGLDNLIVLEIE

>TcasOBP20
MATRFCFGLLILFVGTVLVFAENEHEILEVRALCMNETGVSEETARNYKPAEDPASEEILCM
VKCIFEKIGCLKDDGSFCVDTMKKKNYIMDVINEENEEKIYECLRGVGKITNCRDMAAVEE
CFVKNDSK

>TcasOBP21
MLRLVSLLCLFLLVQGENLDMFDPAGLQACMKKLSVGETELAKALEDKSKDPPEKIMCLFK
CALEDSGFLQDGVVDKSKWPMPECVQDVVKITNCNDMVALKHCFD

>TcasOBP22
MKPIFAIITLTLCTTVHALDCGIHINKNDALKATINKCLISNKTLEDLWDMAPMSSESDSSSE

EVPPVDGKMLQNFRIKRASVRLTNTETNETTPEPKAVSSEAQATENCIIQCIFDNLQMTDST
GYPVHTKILDGLLKNTTNRELRDFLQDTTDECFQVMDKEDTMDPCSYSNKLVTCLAEKGR
SNCADWPVGELPFKP

>TcasOBP23
MKYFPHLCLCLIFFELSEAAMSEAQLKAAVKLVRNMCQPKSKATNEDIEKMHHGDWNID
RTAMCYMHCALNSNKLITKENVFNRDYAITLAEKNLPTALKTASIEAANLCKDSAKTLDD
KCVAAYEISKCLYESNPEKYFLP

>TcasOBP24
MSRMLPAALFVVLATLTFATAEIVVPDDLKDYINELHDHCLKEMGLTEGDHKNYNIHVKD
PKMMCYMKCLMTTSKWMNMDESIQYDFILSSVHPAVKNILLPALDKCRDIPKGTMECEKA
YNFNMCLFNADPENWFFI

>TcasOBP25
MPLKNLIILIVCPLFVFAKVDIPPDLQAEIDGYYDICYKQIGLTKDDLKAYKIGDRDPKIMCF
MKCVFVEAKWMDENENLQYDYIKNTIHHSIRHITLPELENCGKKAEGDKCEKSFSFFNCM
NKAEPEDWVLIQ

>TcasOBP26
MMHLKNFVVLVVCPLFVFAKVEIPPDLEAEIDEYFEQCFEPNGVTMDDIKAYKMGDKDPKI
MCFMRCLFVSGKWMDENENMQYDYIKETIHHAIRHITIPELENCGKEAQTGDKCEKSFNF
FMCMNRAEPEDWILDYKS
